# Implementation of SWAP test for two unknown states in photons via cross-Kerr nonlinearities under decoherence effect

**DOI:** 10.1038/s41598-019-42662-4

**Published:** 2019-04-16

**Authors:** Min-Sung Kang, Jino Heo, Seong-Gon Choi, Sung Moon, Sang-Wook Han

**Affiliations:** 1 Center for Quantum Information, Korea Institute of Science and Technology (KIST), Seoul, 02792 Republic of Korea; 2 College of Electrical and Computer Engineering, Chungbuk National University, Chungdae-ro 1, Seowon-Gu, Cheongju, Republic of Korea

## Abstract

We present an optical scheme for a SWAP test (controlled swap operation) that can determine whether the difference between two unknown states (photons) using cross-Kerr nonlinearities (XKNLs). The SWAP test, based on quantum fingerprinting, has been widely applied to various quantum information processing (QIP) schemes. Thus, for a reliable QIP scheme, it is important to implement a scheme for a SWAP test that is experimentally feasible. Here, we utilize linearly and nonlinearly optical (XKNLs) gates to design a scheme for a SWAP test. We also analyze the efficiency and the performance of nonlinearly optical gates in our scheme under the decoherence effect and exhibit a technique employing quantum bus beams and photon-number-resolving measurements to reduce the effect of photon loss and dephasing caused by the decoherence effect. Consequently, our scheme, which is designed using linearly optical devices and XKNLs (nonlinear optics), can feasibly operate the nearly deterministic SWAP test with high efficiency, in practice.

## Introduction

The development of quantum technology has been explosive. Beyond basic quantum cryptography and quantum computing, new fields such as quantum machine learning^1–4^, quantum communication^5–10^, advanced quantum computing^11–13^, and quantum fingerprinting^14^ have been proposed. A main technique in this area is a SWAP test (controlled swap operation)^14–17^. The SWAP test can determine with certainty whether two unknown states are different^18–20^. Basically, the SWAP test involves a Fredkin gate. The Fredkin gate is a representative multi-qubit gate and has one control qubit and two target qubits for swap operation with each other, resulting from the state of the control qubit. Recently, methods have been proposed to implement a linearly optical SWAP test^15–17,21,22^. In addition, methods for implementing a SWAP test based on nonlinear optics have also been proposed^23–25^.

Also, two kinds of SWAP tests have been proposed: First, destructive SWAP test is equivalent with Hong-Ou-Mandel (HOM) effect using Mach-Zehnder interferometer^15^. No ancillary photon (qubit) is needed to perform the SWAP test, which can determine with certainty whether two unknown states are different. However, after performed the destructive SWAP test between two unknown states, they cannot maintain pre-measured (two unknown) states by directly applying the measurement to unknown states. In this case, we can only obtain information whether two unknown states are different or not. On the other hand, nondestructive SWAP test^17,22–25^ has ancillary system (photon or qubit) for measurement. This SWAP test can be directly applicable from Fredkin gate, which performs the controlled swap operation. And it’s possible to determine whether the difference between two unknown states to conduct the measurement into the ancillary system. Also, if two unknown states are same, two unknown states can be maintained because of no direct measurement regarding to those. By this advantage, although nondestructive SWAP test has difficulties (using linear-^17,21^ or nonlinear optics^23–25^) to experimentally implement in practice, it is an essential element, and can be directly applied to quantum information processing schemes, such as quantum machine learning^1–4^, quantum communication^5–10^, advanced quantum computing^11–13^, and quantum fingerprinting^14^).

To realize a SWAP test using nonlinear optics, the interaction of cross-Kerr nonlinearity (XKNL) can be experimentally implemented in practice. The interactions of XKNL between photons and Kerr media are utilized as a quantum non-demolition measurement, in which the indirect measurement of an ancillary (probe) system is applied to a photon-probe system to acquire the form of the quantum state. Thus, many multi-qubit operations or quantum information processing (QIP) schemes have utilized the XKNL interaction between photons, such as in quantum-controlled gates or computations^4,11,23,26–34^, quantum communications^7,10,35–42^, and the generation and measurement of quantum entanglement^5,6,43–51^. However, the output state from nonlinearly optical gates using XKNLs evolves into a mixed state (decreasing fidelity) because of the decoherence effect (caused by photon loss and dephasing), which consistently occurs in the interaction between photons and Kerr media. Recently, methods that can decrease the decoherence effect have been studied that employ photon-number-resolving (PNR) measurement and quantum bus (qubus) beams with a coherent state having a strong amplitude (probe beam)^26,27,32,52,53^ to reduce the decoherence effect.

In this paper, we present an optical scheme for the SWAP test, which is based on quantum fingerprinting^14^, to certainly determine whether two unknown states are different using nonlinearly optical (path-parity and path-merging) gates and a linearly optical gate (HOM gate). For this assessment (certainty difference in two unknown states), our SWAP test scheme utilizes weak XKNLs, qubus (coherent state) beams, and PNR measurements^32,34,49,51^ for path-parity and path-merging gates, and also the HOM effect^54^ to design a HOM gate using a Mach-Zehnder interferometer. Then, we show the high efficiency and the reliable performance of nonlinearly optical (path-parity and path-merging) gates in our SWAP test through analysis of the fidelities of the output states against the decoherence effect (photon loss and dephasing) when increasing the amplitude of the coherent state (probe beams)^26,27,32^, in practice. Consequently, our SWAP test scheme can feasibly be experimentally implemented with high efficiency and reliable performance, and it is robust against the decoherence effect, as determined by our analysis of nonlinearly optical gates that employ weak XKNLs, qubus beams, and PNR measurements with a strong coherent state.

## Scheme of SWAP test via XKNLs and linearly optical effect

**Figure 1:**
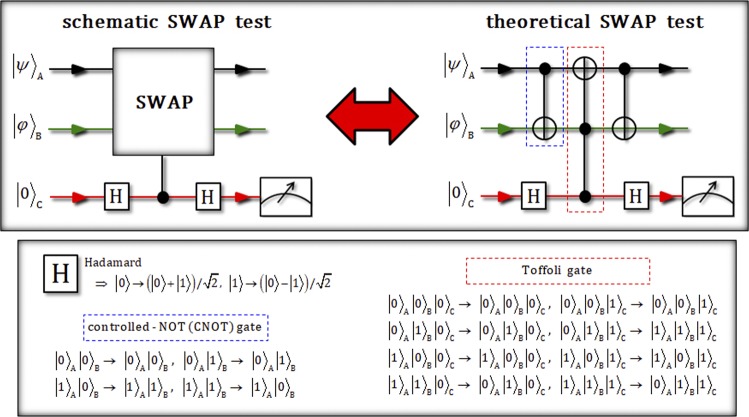
This plot describes a schematic SWAP test and a theoretical SWAP test using CNOT (two-qubit) and Toffoli (three-qubit) gates. The theoretical SWAP test is designed to utilize multi-qubit (two- and three-) controlled gates from the schematic SWAP test, in theory. Actually, the output state from the theoretical SWAP test is the same as the result state of the schematic SWAP test.

First, we introduce the concept of a SWAP test (controlled swap operation) to determine whether two unknown states (|*ψ*〉 and |*φ*〉) are different. Figure 1 shows a schematic SWAP test and a theoretical SWAP test, consisting of two controlled-NOT (CNOT) gates (two-qubit operation) and one Toffoli gate (three-qubit operation)^55^. The two SWAP tests in Fig. 1 are equivalent in terms of the two output states.

Let us assume that the input states are ${|\psi \rangle }_{{\rm{A}}}\equiv \alpha {|0\rangle }_{{\rm{A}}}+\beta {|1\rangle }_{{\rm{A}}}$, ${|\phi \rangle }_{{\rm{B}}}\equiv \delta {|0\rangle }_{{\rm{B}}}+\lambda {|1\rangle }_{{\rm{B}}}$ (two unknown states: we want to distinguish), and |0〉_C_ (control qubit: ancillary qubit), as described in Fig. 1. After the input state, ${|\psi \rangle }_{{\rm{A}}}{|\phi \rangle }_{{\rm{B}}}\otimes {|0\rangle }_{{\rm{C}}}$, passes the sequential operations [Hadamard gates, and controlled swap gates (or CNOT and Toffoli gates in the circuit of the SWAP test)], the result state, pre-measurement, will be given by

1 $$\begin{array}{c}{|\psi \rangle }_{{\rm{A}}}{|\phi \rangle }_{{\rm{B}}}\otimes {|0\rangle }_{{\rm{C}}}\,\mathop{\to }\limits^{\boxed{\displaystyle H}}\,{|\psi \rangle }_{{\rm{A}}}{|\phi \rangle }_{{\rm{B}}}\otimes ({|0\rangle }_{{\rm{C}}}+{|1\rangle }_{{\rm{C}}})/\sqrt{2}\\ \mathop{\longrightarrow }\limits^{{\rm{c}}{\rm{o}}{\rm{n}}{\rm{t}}{\rm{r}}{\rm{o}}{\rm{l}}{\rm{l}}{\rm{e}}{\rm{d}}\,{\rm{s}}{\rm{w}}{\rm{a}}{\rm{p}}({\rm{o}}{\rm{r}}\,{\rm{C}}{\rm{N}}{\rm{O}}{\rm{T}}\to {\rm{T}}{\rm{o}}{\rm{f}}{\rm{f}}{\rm{o}}{\rm{l}}{\rm{i}}\to {\rm{C}}{\rm{N}}{\rm{O}}{\rm{T}})}\\ \to ({|\psi \rangle }_{{\rm{A}}}{|\phi \rangle }_{{\rm{B}}}\otimes {|0\rangle }_{{\rm{C}}}+{|\phi \rangle }_{{\rm{A}}}{|\psi \rangle }_{{\rm{B}}}\otimes {|1\rangle }_{{\rm{C}}})/\sqrt{2}\\ \mathop{\to }\limits^{\boxed{\displaystyle H}}[({|\psi \rangle }_{{\rm{A}}}{|\phi \rangle }_{{\rm{B}}}+{|\phi \rangle }_{{\rm{A}}}{|\psi \rangle }_{{\rm{B}}})\otimes {|0\rangle }_{{\rm{C}}}+({|\psi \rangle }_{{\rm{A}}}{|\phi \rangle }_{{\rm{B}}}-{|\phi \rangle }_{{\rm{A}}}{|\psi \rangle }_{{\rm{B}}})\otimes {|1\rangle }_{{\rm{C}}}]/2.\end{array}$$

When the ancillary qubit, C, is measured, we can determine that two unknown states, A and B, are identical or not, according to Eq. 1. If the two unknown state are the same (${|\psi \rangle }_{{\rm{A}}}={|\phi \rangle }_{{\rm{B}}}$), the result of measurement in the ancillary state is |0〉_C_ with probability 1 because the result state is ${|\psi \rangle }_{{\rm{A}}}{|\phi \rangle }_{{\rm{B}}}\otimes {|0\rangle }_{{\rm{C}}}$. In another case, ${|\psi \rangle }_{{\rm{A}}}\ne {|\phi \rangle }_{{\rm{B}}}$, the probabilities of the result state in |0〉_C_ and |1〉_C_ of the ancillary qubit are $(1+{|\langle \varphi |\psi \rangle |}^{2})/2$ and $(1-{|\langle \varphi |\psi \rangle |}^{2})/2$ from Eq. 1, respectively. Thus, if the result of the ancillary qubit is in state |1〉_C_, we can be convinced that two unknown states are different. Consequently, we can determine the result of the difference in the two unknown states with reliability through the SWAP test, in principle.

To determine the performance property of nonlinearly optical (path-parity and path-merging) gates using XKNLs, we introduce the Hamiltonian, *H*_*Kerr*_, of the XKNLS effect (*H*_*Kerr*_ = *ℏχN*_1_*N*_2_ for *N*_*i*_: photon number operator, and *χ*: strength of nonlinearity in a Kerr medium). The unitary operation^26–51^ of the XKNL is expressed as ${{\rm{U}}}_{Kerr}{|n\rangle }_{1}{|\alpha \rangle }_{2}={e}^{it{H}_{Kerr}/\hslash }{|n\rangle }_{1}{|\alpha \rangle }_{2}={e}^{i\theta {N}_{1}{N}_{2}}{|n\rangle }_{1}{|\alpha \rangle }_{2}={|n\rangle }_{1}{|\alpha {e}^{in\theta }\rangle }_{2}$ between the photon (|*n*〉_1_: photon number state) and the coherent state (|*α*〉_2_: probe beam), where *θ* ( = *χt*) is the magnitude of the conditional phase shift caused by XKNL, and *t* is the interaction time in a Kerr medium.

**Figure 2:**
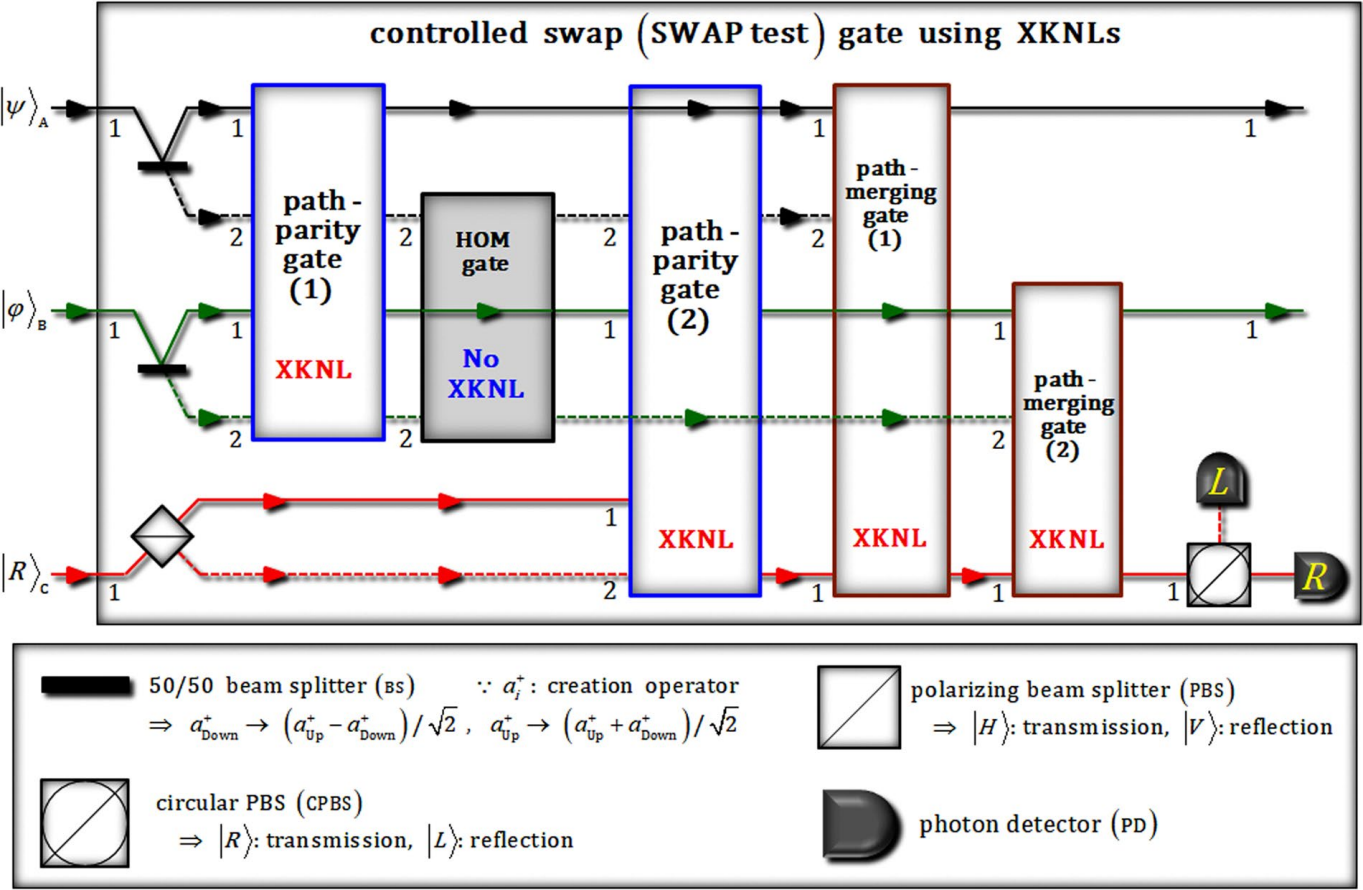
Schematic plot of SWAP test (controlled swap gate): This scheme consists of two path-parity gates (1 and 2) and two path-merging gates (1 and 2) using XKNLs, and an HOM gate using the HOM effect with linearly optical devices. As a result of the outcome of measurement of photon C (ancillary photon), this scheme (SWAP test) can make a comparison to determine if two unknown states of photons (A and B) are different. Multi-qubit gates via XKNLs are utilized in our SWAP test for experimental realization.

From now on, we propose an optical scheme of the SWAP test to be implemented using XKNLs (nonlinear optics) and the HOM effect (linear optics), as described in Fig. 2. We assume two unknown states (A and B) of photons, and an ancillary photon (C: control qubit), as ${|\psi \rangle }_{{\rm{A}}}\equiv \alpha {|H\rangle }_{{\rm{A}}}+\beta {|V\rangle }_{{\rm{A}}}$ and ${|\phi \rangle }_{{\rm{B}}}\equiv \delta {|H\rangle }_{{\rm{B}}}+\lambda {|V\rangle }_{{\rm{B}}}$, and |*R*〉_C_, where the circular polarization (|*R*〉: right, |*L*〉: left) are related to the linear polarization (|*H*〉: horizontal, |*V*〉: vertical) with the relationship $|R\rangle \equiv (|H\rangle +|V\rangle )/\sqrt{2}$ and $|L\rangle \equiv (|H\rangle -|V\rangle )/\sqrt{2}$. As described in Fig. 2, after this input state, ${|\psi \rangle }_{{\rm{A}}}^{1}{|\phi \rangle }_{{\rm{B}}}^{1}\otimes {|R\rangle }_{{\rm{C}}}^{1}$, passes through two BSs and a CPBS, the state, |Φ_1_〉_ABC_, is transformed as

2 $$\begin{array}{c}{|\psi \rangle }_{{\rm{A}}}^{1}{|\phi \rangle }_{{\rm{B}}}^{1}\otimes {|R\rangle }_{{\rm{C}}}^{1}\mathop{\to }\limits^{{\rm{B}}{\rm{S}}{\rm{s}},{\rm{C}}{\rm{P}}{\rm{B}}{\rm{S}}}\\ \to {|{{\rm{\Phi }}}_{1}\rangle }_{{\rm{A}}{\rm{B}}{\rm{C}}}=\frac{1}{2}({|\psi \rangle }_{{\rm{A}}}^{1}{|\phi \rangle }_{{\rm{B}}}^{1}+{|\psi \rangle }_{{\rm{A}}}^{1}{|\phi \rangle }_{{\rm{B}}}^{2}+{|\psi \rangle }_{{\rm{A}}}^{2}{|\phi \rangle }_{{\rm{B}}}^{1}+{|\psi \rangle }_{{\rm{A}}}^{2}{|\phi \rangle }_{{\rm{B}}}^{2})\otimes \frac{1}{\sqrt{2}}({|H\rangle }_{{\rm{C}}}^{1}+{|V\rangle }_{{\rm{C}}}^{2}).\end{array}$$

**Figure 3:**
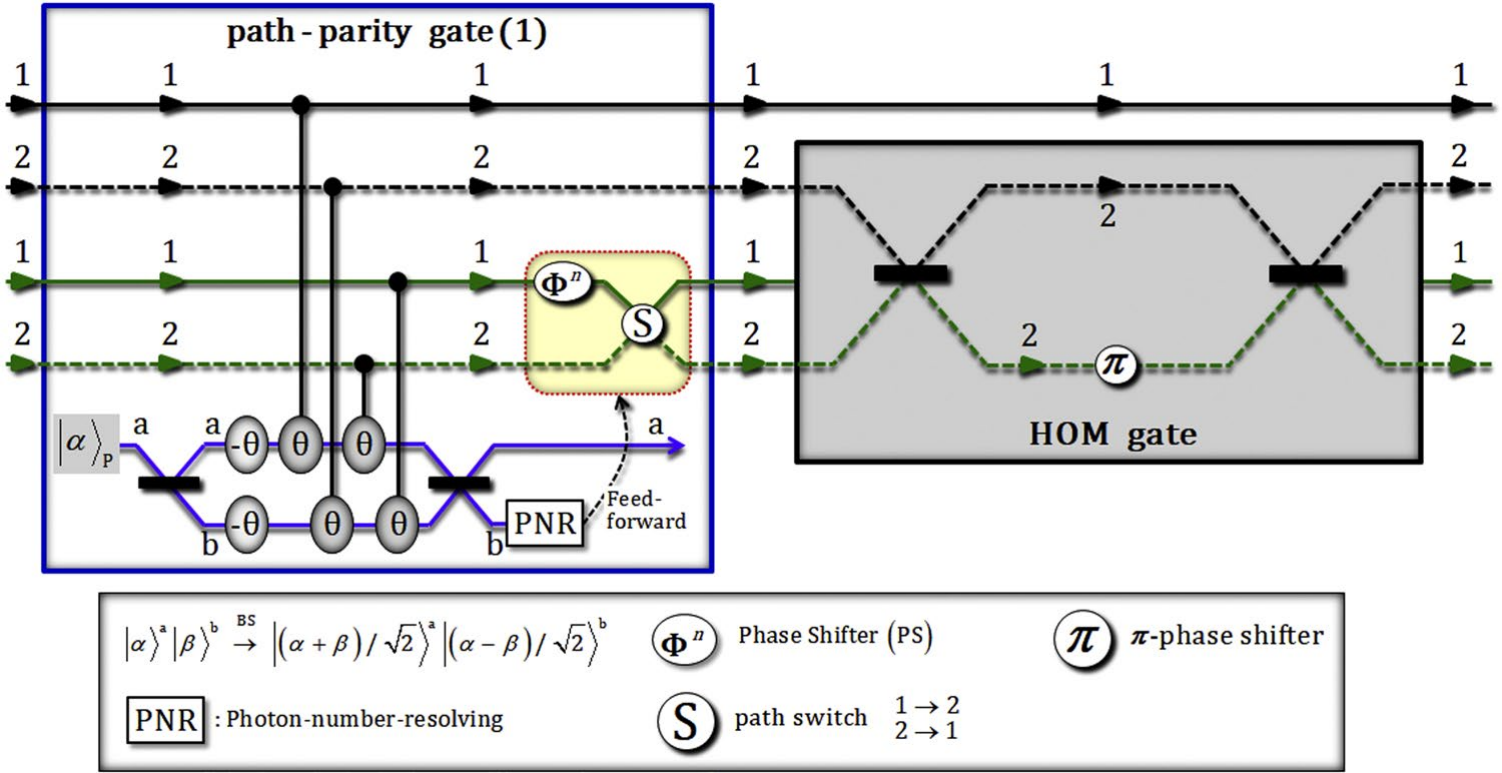
[path-parity gate (1)] - This gate consists of weak XKNLs, qubus beams, and PNR measurement. After PNR measurement in a qubus beam (path b), the feed-forward process is applied to photon B as a result of the outcome of PNR measurement. The output state from this gate is transformed to the form that has the same paths (the sorted paths) of photons A and B. [HOM gate] - This gate is composed of a Mach-Zehnder interferometer with a *π*– phase shifter and employs the HOM effect^54^. The output states (photons A and B) from this gate are swapped by passing through two BSs and a *π*– phase shifter.

Then, two photons (A and B) in this state, |Φ_1_〉_ABC_, are injected to the path-parity gate (1) using XKNLs, qubus beams, and PNR measurement, as described in Fig. 3.

After the operation on the state |Φ_1_〉_ABC_ in Fig. 3, the state, ${|{{\rm{\Phi }}}_{2}\rangle }_{{\rm{CAB}}\otimes {\rm{P}}}$ (pre-measurement) from path-parity gate (1) is expressed as

3 $$\begin{array}{c}{|{{\rm{\Phi }}}_{1}\rangle }_{{\rm{A}}{\rm{B}}{\rm{C}}}\mathop{\to }\limits^{{\rm{p}}{\rm{a}}{\rm{t}}{\rm{h}}-{\rm{p}}{\rm{a}}{\rm{r}}{\rm{i}}{\rm{t}}{\rm{y}}\,{\rm{g}}{\rm{a}}{\rm{t}}{\rm{e}}(1)}\\ \begin{array}{ccc}\to {|{{\rm{\Phi }}}_{2}\rangle }_{{\rm{C}}{\rm{A}}{\rm{B}}\otimes {\rm{P}}} & = & \frac{1}{\sqrt{2}}[\{\frac{1}{\sqrt{2}}({|H\rangle }_{{\rm{C}}}^{1}+{|V\rangle }_{{\rm{C}}}^{2})\otimes \frac{1}{\sqrt{2}}({|\psi \rangle }_{{\rm{A}}}^{1}{|\phi \rangle }_{{\rm{B}}}^{1}+{|\psi \rangle }_{{\rm{A}}}^{2}{|\phi \rangle }_{{\rm{B}}}^{2})\}\\  &  & \otimes \,{|\alpha \rangle }_{{\rm{P}}}^{{\rm{a}}}{|0\rangle }_{{\rm{P}}}^{{\rm{b}}}+{e}^{-\frac{{(\alpha \sin \theta )}^{2}}{2}}\,\sum _{n=0}^{{\rm{\infty }}}\frac{{(i\alpha \sin \theta )}^{n}}{\sqrt{n!}}\,\{\frac{1}{\sqrt{2}}({|H\rangle }_{{\rm{C}}}^{1}+{|V\rangle }_{{\rm{C}}}^{2})\\  &  & \otimes \,\frac{1}{\sqrt{2}}({|\psi \rangle }_{{\rm{A}}}^{1}{|\phi \rangle }_{{\rm{B}}}^{2}+{(-1)}^{n}{|\psi \rangle }_{{\rm{A}}}^{2}{|\phi \rangle }_{{\rm{B}}}^{1})\}\otimes \,{|\alpha \,\cos \,\theta \rangle }_{{\rm{P}}}^{{\rm{a}}}{|n\rangle }_{{\rm{P}}}^{{\rm{b}}}],\end{array}\end{array}$$

where $$|\pm i\alpha \,\sin \,\theta \rangle ={e}^{-\frac{{(\alpha \sin \theta )}^{2}}{2}}\sum _{n=0}^{\infty }\frac{{(\pm i\alpha \sin \theta )}^{n}}{\sqrt{n!}}|n\rangle $$ for α ∈ **R**. When the PNR measurement [For precisely measuring photon number, we use the quantum non-demolition detection^28,29,31,41^ using positive-operator-value measurement (POVM) elements: APPENDIX (A)] is applied in the coherent state (probe beam) of path b, if the outcome is dark detection, $${|0\rangle }_{{\rm{P}}}^{{\rm{b}}}$$, the output state, |Φ_2_〉_CAB_, is acquired as $${|{{\rm{\Phi }}}_{2}\rangle }_{{\rm{CAB}}}=({|H\rangle }_{{\rm{C}}}^{1}+{|V\rangle }_{{\rm{C}}}^{2})/\sqrt{2}\otimes $$$$({|\psi \rangle }_{{\rm{A}}}^{1}{|\phi \rangle }_{{\rm{B}}}^{1}+{|\psi \rangle }_{{\rm{A}}}^{2}{|\phi \rangle }_{{\rm{B}}}^{2})/\sqrt{2}$$. Also, if the result is the state $${|n\rangle }_{{\rm{P}}}^{{\rm{b}}}$$ (*n* ≠ 0), the output state can be transformed to the state |Φ_2_〉_CAB_ (dark detection) by feed-forward [PS, and path switch: APPENDIX (B)] in terms of the result (photon number *n*) on path b. Subsequently, the states of photons (A and B) on path 2 in the state |Φ_2_〉_CAB_ will be exchanged (swapped) to the state |Φ_3_〉_CAB_ after passing through the HOM gate, as follows:

4 $${|{{\rm{\Phi }}}_{2}\rangle }_{{\rm{CAB}}}\mathop{\to }\limits^{{\rm{HOM}}\,{\rm{gate}}}{|{{\rm{\Phi }}}_{3}\rangle }_{{\rm{CAB}}}=\frac{1}{\sqrt{2}}({|H\rangle }_{{\rm{C}}}^{1}+{|V\rangle }_{{\rm{C}}}^{2})\otimes \frac{1}{\sqrt{2}}({|\psi \rangle }_{{\rm{A}}}^{1}{|\phi \rangle }_{{\rm{B}}}^{1}+{|\phi \rangle }_{{\rm{A}}}^{2}{|\psi \rangle }_{{\rm{B}}}^{2}),$$

where the HOM gate (linear optics) using the HOM effect^54^, in Fig. 3, performs the swap operation. Consequently, the output state, |Φ_3_〉_CAB_ is transformed to the form (Eq. 4) having the same path (1 or 2) between two photons (A and B) by path-parity gate (1), and also the state of the two photons on path 2 are swapped by the HOM gate.

**Figure 4:**
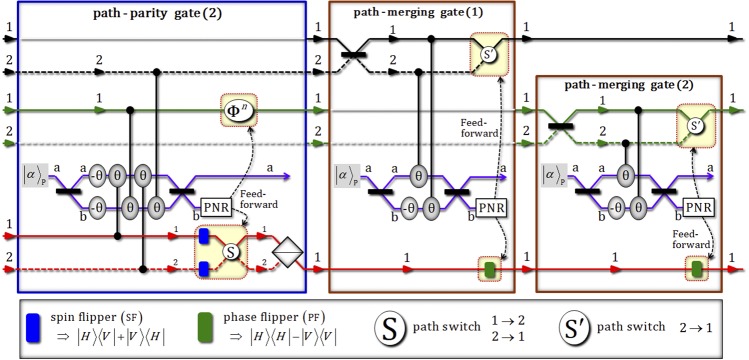
[path-parity gate (2)] - This gate consists of weak XKNLs, qubus beams, and PNR measurement. After PNR measurement in a qubus beam (path b), the process of feed-forward is applied to photons (B and C) as a result of the outcome of PNR measurement. The output state from this gate is transformed to the form that has the same paths (the sorted paths) of photons A, B, and C, before PBS on photon C. [path-merging gates (1 and 2)] - These gates are composed of weak XKNLs, qubus beams, PNR measurements (on path b), and feed-forwards. The spilt paths (1 and 2) of photons A and B are merged into one path 1 by the operations of path-merging gates (1 and 2), respectively.

Then, three photons (A, B, and C) in this state, |Φ_3_〉_CAB_, pass through path-parity gate (2) using XKNLs, qubus beams, and PNR measurement, as described in Fig. 4. After the operation, shown in Fig. 4, of path-parity gate (2) on the state |Φ_3_〉_CAB_, the state, ${|{{\rm{\Phi }}}_{4}\rangle }_{{\rm{CAB}}\otimes {\rm{P}}}$ (pre-measurement), is given by

5 $$\begin{array}{c}{|{{\rm{\Phi }}}_{3}\rangle }_{{\rm{C}}{\rm{A}}{\rm{B}}}\mathop{\to }\limits^{{\rm{p}}{\rm{a}}{\rm{t}}{\rm{h}}-{\rm{p}}{\rm{a}}{\rm{r}}{\rm{i}}{\rm{t}}{\rm{y}}\,{\rm{g}}{\rm{a}}{\rm{t}}{\rm{e}}(2)}\\ \begin{array}{ccc}\to {|{{\rm{\Phi }}}_{4}\rangle }_{{\rm{C}}{\rm{A}}{\rm{B}}\otimes {\rm{P}}} & = & \frac{1}{\sqrt{2}}[\frac{1}{\sqrt{2}}({|H\rangle }_{{\rm{C}}}^{1}\otimes {|\psi \rangle }_{{\rm{A}}}^{1}{|\phi \rangle }_{{\rm{B}}}^{1}+\,{|V\rangle }_{{\rm{C}}}^{2}\otimes {|\phi \rangle }_{{\rm{A}}}^{2}{|\psi \rangle }_{{\rm{B}}}^{2})\otimes {|\alpha \rangle }_{{\rm{P}}}^{{\rm{a}}}{|0\rangle }_{{\rm{P}}}^{{\rm{b}}}\\  &  & +\,{e}^{-\frac{{(\alpha \sin \theta )}^{2}}{2}}\sum _{n=0}^{{\rm{\infty }}}\frac{{(i\alpha \sin \theta )}^{n}}{\sqrt{n!}}\{\frac{1}{\sqrt{2}}({|H\rangle }_{{\rm{C}}}^{1}\otimes {|\phi \rangle }_{{\rm{A}}}^{2}{|\psi \rangle }_{{\rm{B}}}^{2}\\  &  & +\,{(-1)}^{n}{|V\rangle }_{{\rm{C}}}^{2}\otimes {|\psi \rangle }_{{\rm{A}}}^{1}{|\phi \rangle }_{{\rm{B}}}^{1})\otimes \,{|\alpha \,\cos \,\theta \rangle }_{{\rm{P}}}^{{\rm{a}}}{|n\rangle }_{{\rm{P}}}^{{\rm{b}}}].\end{array}\end{array}$$

When the PNR measurement is applied in the coherent state (probe beam) of path b, if the outcome is dark detection, ${|0\rangle }_{{\rm{P}}}^{{\rm{b}}}$, the output state, |Φ_4_〉_CAB_, is acquired as ${|{{\rm{\Phi }}}_{4}\rangle }_{{\rm{CAB}}}=({|H\rangle }_{{\rm{C}}}^{1}\otimes {|\psi \rangle }_{{\rm{A}}}^{1}{|\phi \rangle }_{{\rm{B}}}^{1}+{|V\rangle }_{{\rm{C}}}^{2}\otimes {|\phi \rangle }_{{\rm{A}}}^{2}{|\psi \rangle }_{{\rm{B}}}^{2})/\sqrt{2}$. Also, if the result is the state ${|n\rangle }_{{\rm{P}}}^{{\rm{b}}}$ (*n* ≠ 0), the output state can be transformed to the state |Φ_4_〉_CAB_ (dark detection) by feed-forward (PS, SF, and path switch) with regard to the result (photon number *n*) on path b.

Then, after the photon C in the state |Φ_4_〉_CAB_ passes through PBS in path-parity gate (2), the output state, |Φ_5_〉_ABC_, is expressed as

6 $${|{{\rm{\Phi }}}_{4}\rangle }_{{\rm{CAB}}}\mathop{\to }\limits^{{\rm{PBS}}}{|{{\rm{\Phi }}}_{5}\rangle }_{{\rm{ABC}}}=\frac{1}{\sqrt{2}}({|\psi \rangle }_{{\rm{A}}}^{1}{|\phi \rangle }_{{\rm{B}}}^{1}\otimes {|H\rangle }_{{\rm{C}}}^{1}+{|\phi \rangle }_{{\rm{A}}}^{2}{|\psi \rangle }_{{\rm{B}}}^{2}\otimes {|V\rangle }_{{\rm{C}}}^{1}).$$

Subsequently, for the merging paths (1 and 2) of photon A in Eq. 6, the state |Φ_5_〉_ABC_ passes through path-merging gate (1). After the operation, shown in Fig. 4, of path-merging gate (1) on the state |Φ_5_〉_ABC_, the state, ${|{{\rm{\Phi }}}_{6}\rangle }_{{\rm{ABC}}\otimes {\rm{P}}}$ (pre-measurement), is given by

7 $$\begin{array}{c}{|{{\rm{\Phi }}}_{5}\rangle }_{{\rm{A}}{\rm{B}}{\rm{C}}}\mathop{\to }\limits^{{\rm{m}}{\rm{e}}{\rm{r}}{\rm{g}}{\rm{i}}{\rm{n}}{\rm{g}}-{\rm{p}}{\rm{a}}{\rm{r}}{\rm{i}}{\rm{t}}{\rm{y}}\,{\rm{g}}{\rm{a}}{\rm{t}}{\rm{e}}(1)}\\ \begin{array}{ccc}\to {|{{\rm{\Phi }}}_{6}\rangle }_{{\rm{A}}{\rm{B}}{\rm{C}}\otimes {\rm{P}}} & = & \frac{1}{\sqrt{2}}[\frac{1}{\sqrt{2}}({|\psi \rangle }_{{\rm{A}}}^{1}{|\phi \rangle }_{{\rm{B}}}^{1}\otimes {|H\rangle }_{{\rm{C}}}^{1}\,+\,{|\phi \rangle }_{{\rm{A}}}^{1}{|\psi \rangle }_{{\rm{B}}}^{2}\otimes {|V\rangle }_{{\rm{C}}}^{1})\otimes \,{|\alpha \rangle }_{{\rm{P}}}^{{\rm{a}}}{|0\rangle }_{{\rm{P}}}^{{\rm{b}}}\\  &  & +\,{e}^{-\frac{{(\alpha \sin \theta )}^{2}}{2}}\,\sum _{n=0}^{{\rm{\infty }}}\frac{{(i\alpha \sin \theta )}^{n}}{\sqrt{n!}}\{\frac{1}{\sqrt{2}}({|\psi \rangle }_{{\rm{A}}}^{2}{|\phi \rangle }_{{\rm{B}}}^{1}\otimes {|H\rangle }_{{\rm{C}}}^{1}\\  &  & -\,{|\phi \rangle }_{{\rm{A}}}^{2}{|\psi \rangle }_{{\rm{B}}}^{2}\otimes {|V\rangle }_{{\rm{C}}}^{1})\}\otimes {|\alpha \,\cos \,\theta \rangle }_{{\rm{P}}}^{{\rm{a}}}{|n\rangle }_{{\rm{P}}}^{{\rm{b}}}].\end{array}\end{array}$$

According to the result of PNR measurement in the coherent state (probe beam) of path b, the output state is obtained as ${|{{\rm{\Phi }}}_{6}\rangle }_{{\rm{ABC}}}=({|\psi \rangle }_{{\rm{A}}}^{1}{|\phi \rangle }_{{\rm{B}}}^{1}\otimes {|H\rangle }_{{\rm{C}}}^{1}+{|\phi \rangle }_{{\rm{A}}}^{1}{|\psi \rangle }_{{\rm{B}}}^{2}\otimes {|V\rangle }_{{\rm{C}}}^{1})/\sqrt{2}$ without feed-forward or with feed-forward (PF and path switch). Also, as described in Fig. 4, the state |Φ_6_〉_ABC_ will be expressed as ${|{{\rm{\Phi }}}_{7}\rangle }_{{\rm{ABC}}\otimes {\rm{P}}}$ (pre-measurement) after the path-merging gate (2) regarding photon B, as follows:

8 $$\begin{array}{c}{|{{\rm{\Phi }}}_{6}\rangle }_{{\rm{A}}{\rm{B}}{\rm{C}}}\mathop{\to }\limits^{{\rm{m}}{\rm{e}}{\rm{r}}{\rm{g}}{\rm{i}}{\rm{n}}{\rm{g}}-{\rm{p}}{\rm{a}}{\rm{r}}{\rm{i}}{\rm{t}}{\rm{y}}\,{\rm{g}}{\rm{a}}{\rm{t}}{\rm{e}}(2)}\\ \begin{array}{ccc}\to {|{{\rm{\Phi }}}_{7}\rangle }_{{\rm{A}}{\rm{B}}{\rm{C}}\otimes {\rm{P}}} & = & \frac{1}{\sqrt{2}}[\frac{1}{\sqrt{2}}({|\psi \rangle }_{{\rm{A}}}^{1}{|\phi \rangle }_{{\rm{B}}}^{1}\otimes {|H\rangle }_{{\rm{C}}}^{1}+\,{|\phi \rangle }_{{\rm{A}}}^{1}{|\psi \rangle }_{{\rm{B}}}^{1}\otimes {|V\rangle }_{{\rm{C}}}^{1})\otimes {|\alpha \rangle }_{{\rm{P}}}^{{\rm{a}}}{|0\rangle }_{{\rm{P}}}^{{\rm{b}}}\\  &  & +\,{e}^{-\frac{{(\alpha \sin \theta )}^{2}}{2}}\sum _{n=0}^{{\rm{\infty }}}\frac{{(i\alpha \sin \theta )}^{n}}{\sqrt{n!}}\{\frac{1}{\sqrt{2}}({|\psi \rangle }_{{\rm{A}}}^{1}{|\phi \rangle }_{{\rm{B}}}^{2}\otimes {|H\rangle }_{{\rm{C}}}^{1}\\  &  & -\,{|\phi \rangle }_{{\rm{A}}}^{1}{|\psi \rangle }_{{\rm{B}}}^{2}\otimes {|V\rangle }_{{\rm{C}}}^{1})\}\otimes {|\alpha \,\cos \,\theta \rangle }_{{\rm{P}}}^{{\rm{a}}}{|n\rangle }_{{\rm{P}}}^{{\rm{b}}}].\end{array}\end{array}$$

Then, through the PNR measurement and feed-forward (PF and path switch) in path-merging gate (2), the output state is given by

9 $${|{{\rm{\Phi }}}_{7}\rangle }_{{\rm{ABC}}}=\frac{1}{\sqrt{2}}({|\psi \rangle }_{{\rm{A}}}^{1}{|\phi \rangle }_{{\rm{B}}}^{1}\otimes {|H\rangle }_{{\rm{C}}}^{1}+{|\phi \rangle }_{{\rm{A}}}^{1}{|\psi \rangle }_{{\rm{B}}}^{1}\otimes {|V\rangle }_{{\rm{C}}}^{1}).$$

From the input state, ${|\psi \rangle }_{{\rm{A}}}^{1}{|\phi \rangle }_{{\rm{B}}}^{1}\otimes {|R\rangle }_{{\rm{C}}}^{1}$, this output state, |Φ_7_〉_ABC_, in Eq. 9 is transformed by passing the nonlinearly and nearly optical gates (path-parity, path-merging, and HOM gates). Finally, the final state, |Φ_f_〉_ABC_, is the same as the output state of the SWAP test in Fig. 1 after CPBS operates on photon C of the output state, |Φ_7_〉_ABC_, as follows:

10 $$\begin{array}{c}{|{{\rm{\Phi }}}_{7}\rangle }_{{\rm{A}}{\rm{B}}{\rm{C}}}\mathop{\to }\limits^{{\rm{C}}{\rm{P}}{\rm{B}}{\rm{S}}}\\ \to {|{{\rm{\Phi }}}_{{\rm{f}}}\rangle }_{{\rm{A}}{\rm{B}}{\rm{C}}}=\frac{1}{\sqrt{2}}[({|\psi \rangle }_{{\rm{A}}}^{1}{|\phi \rangle }_{{\rm{B}}}^{1}+{|\phi \rangle }_{{\rm{A}}}^{1}{|\psi \rangle }_{{\rm{B}}}^{1})\otimes {|R\rangle }_{{\rm{C}}}+({|\psi \rangle }_{{\rm{A}}}^{1}{|\phi \rangle }_{{\rm{B}}}^{1}-{|\phi \rangle }_{{\rm{A}}}^{1}{|\psi \rangle }_{{\rm{B}}}^{1})\otimes {|L\rangle }_{{\rm{C}}}].\end{array}$$

Consequently, we can determine that two unknown states, A and B, are identical or not through the final state |Φ_f_〉_ABC_, in Eq. 10, which is generated by our optical scheme in Fig. 2. In our schematic SWAP test, the nonlinearly optical gates (two path-parity and two path-merging gates) are critical components for implementing the SWAP test. Thus, to ensure the high efficiency of these gates, the error probabilities (${{\rm{P}}}_{{\rm{err}}}^{{\rm{P}}}$: path-parity gate and ${{\rm{P}}}_{{\rm{err}}}^{{\rm{M}}}$: path-merging gate) can be estimated by the probability to measure ${|0\rangle }_{{\rm{P}}}^{{\rm{b}}}$ (dark detection) in ${|\pm i\alpha \,\sin \,\theta \rangle }_{{\rm{P}}}^{{\rm{b}}}$ on path b of the qubus beams (Figs 3 and 4), as follows:

11 $${{\rm{P}}}_{{\rm{err}}}^{{\rm{P}}}={{\rm{P}}}_{{\rm{err}}}^{{\rm{M}}}=\frac{1}{2}\exp (-{\alpha }^{2}{\sin }^{2}\theta )\approx \frac{1}{2}\exp (-{\alpha }^{2}{\theta }^{2}),$$

where ${\alpha }^{2}{\sin }^{2}\theta \approx {\alpha }^{2}{\theta }^{2}$ for *α* ≫ 1 *θ* ≪ 1 and. If the parameters (*α*: amplitude of coherent state and *θ*: magnitude of conditional phase shift) are fixed as *αθ* = 2.5, the error probabilities (${{\rm{P}}}_{{\rm{err}}}^{{\rm{P}}}$ and ${{\rm{P}}}_{{\rm{err}}}^{{\rm{M}}}$) can be acquired as ${{\rm{P}}}_{{\rm{err}}}^{{\rm{P}}}={{\rm{P}}}_{{\rm{err}}}^{{\rm{M}}} < {10}^{-3}$. Moreover, when we increase the amplitude of the coherent state or magnitude of the conditional phase shift in nonlinearly optical gates, the error probabilities (${{\rm{P}}}_{{\rm{err}}}^{{\rm{P}}}$ and ${{\rm{P}}}_{{\rm{err}}}^{{\rm{M}}}$) can approach zero.

So far, we have presented an optical scheme to implement a SWAP test using nonlinearly optical gates (XKNLs, qubus beams, and PNR measurement) and a linearly optical gate (HOM gate) to determine if two unknown states are identical or not. However, because of the use of XKNLs in our scheme, the decoherence effect (photon loss and dephasing), which can induce the evolution of a quantum pure state into a mixed state, occurs in nonlinearly optical gates (path-parity and path-merging gates) when our scheme is experimentally realized in practical optical fibers^56,57^. Thus, we propose a method^26,27,32^ for the nonlinearly optical gates (via XKNLs, qubus beams, and PNR measurement) to obtain robustness against the decoherence effect.

## Analysis of path-parity and path-merging gates under decoherence effect

The nonlinearly optical (path-parity and path-merging) gates consist of the interactions of XKNLs, qubus beams (coherent state), and PNR measurements and are essential components for implementing the proposed SWAP test (controlled swap operation) scheme. However, in optical fibers^56,57^, photon loss (increasing error probability) in the probe beam and dephasing coherent parameters in the photon-probe system (decreasing the fidelity of the output state) occur because of the decoherence effect^26,27,32,52,53^ when nonlinearly optical (path-parity and path-merging) gates are implemented in our SWAP test scheme, in practice. Thus, we need to analyze the efficiency (related to photon loss) and performance (related to dephasing) of nonlinearly optical gates, using XKNL, under the decoherence effect, and we also should demonstrate path-parity and path-merging gates, in our scheme, having high efficiency and high fidelity (performance) against the decoherence effect by the utilization of a coherent state with a large amplitude^26,27,32^.

We introduce the solution of the master equation^58^, which can describe the open quantum system (nonunitary operation), for analysis of the decoherence effect in a Kerr medium, as follows:

12 $$\begin{array}{l}\frac{\partial \rho (t)}{\partial t}=-\frac{i}{\hslash }[H,\,\rho ]+\gamma ({a}\rho {{a}}^{+}+\frac{1}{2}({{a}}^{+}{a}\rho +\rho {{a}}^{+}{a})),\\ \because \hat{J}\rho =\gamma {a}\rho {{a}}^{+},\,\hat{L}\rho =-\frac{\gamma }{2}({{a}}^{+}{a}\rho +\rho {{a}}^{+}{a})\end{array}$$

where *γ*, *t* (=*θ*/*χ*), and *a*^+^(*a*) are the energy decay rate, the interaction time, and the creation (annihilation) operator. The solution of the master equation can be written as $\rho (t)=\exp [(\hat{J}+\hat{L})t]\rho (0)$^58^.

For application in the analysis of nonlinearly optical (path-parity and path-merging) gates, we show the process model^26,27,32^ of the interaction of XKNLs and the decoherence effect (photon loss and dephasing) using the solution from the master equation (Eq. 12). We assume that the initial state (photon-probe system) is $|H\rangle \langle V|\otimes |\alpha \rangle \langle \alpha |$, and the interaction of XKNL (conditional phase shift: ${{\rm{U}}}_{Kerr}|H\rangle |\alpha \rangle \to |H\rangle |\alpha {e}^{i\theta }\rangle$) can be operated on the probe beam (coherent state) if the control photon’s polarization is *H* (horizontal). After the interaction of XKNL, ${\tilde{X}}_{t}$, and the decoherence effect, ${\tilde{D}}_{t}$, which can be described as ${\tilde{D}}_{t}|\alpha \rangle \langle \beta |=\exp [-(1-{e}^{-\gamma t})\{-\alpha {\beta }^{\ast }+({|\alpha |}^{2}+{|\beta |}^{2})/2\}]|{{\rm{\Lambda }}}_{t}\alpha \rangle \langle {{\rm{\Lambda }}}_{t}\beta |$, for interaction time *t* (=*θ*/*χ*), the output state can be represented by the solution of the master equation, as follows:

13 $$({\tilde{D}}_{t}{\tilde{X}}_{t})|H\rangle \langle V|\otimes |\alpha \rangle \langle \alpha |=\exp [-{\alpha }^{2}(1-{e}^{-\gamma t})(1-{e}^{i\theta })]|H\rangle \langle V|\otimes |{{\rm{\Lambda }}}_{t}\alpha {e}^{i\theta }\rangle \langle {{\rm{\Lambda }}}_{t}\alpha |,$$

where Λ_*t*_ = *e*^−*γt*/2^ is the rate of remaining photons resulting from photon loss. The coefficient on the right hand side in Eq. 14 is the coherent parameter, which can quantify the degree of dephasing. Note that the operation of the decoherence effect, ${\tilde{D}}_{t}$ occurs with the interaction of XKNL, ${\tilde{X}}_{t}$, in this process. For a good approximation of the process model of the interaction of XKNLs and the decoherence effect, we can take an arbitrarily small time, Δ*t* (=*t*/*N*^[)26,27,32^, for the interaction of XKNL between photons and probe beam in a Kerr medium. Finally, equation 14 can be transformed to the process model^26,27,32^ to analyze the efficiency and performance of nonlinearly optical (path-parity and path-merging) gates, as follows:

14 $${({\tilde{D}}_{{\rm{\Delta }}t}{\tilde{X}}_{{\rm{\Delta }}t})}^{N}|H\rangle \langle V|\otimes |\alpha \rangle \langle \alpha |=\exp [-{\alpha }^{2}(1-{e}^{-\gamma {\rm{\Delta }}t})\sum _{n=1}^{N}{e}^{-\gamma {\rm{\Delta }}t(n-1)}(1-{e}^{in{\rm{\Delta }}\theta })]|H\rangle \langle V|\otimes |{{\rm{\Lambda }}}_{t}\alpha {e}^{i\theta }\rangle \langle {{\rm{\Lambda }}}_{t}\alpha |,$$

where ${\tilde{D}}_{t}{\tilde{X}}_{t}={({\tilde{D}}_{{\rm{\Delta }}t}{\tilde{X}}_{{\rm{\Delta }}t})}^{N}$, and *θ* = *χt* = *χN*Δ*t* = *N*Δ*θ* for small time, Δ*t* (=*t*/*N*), and α ∈ **R**. Also, an optical fiber, in which the nonlinearly optical gate using XKNLs is realized, of approximately 3000 km is required to acquire the magnitude of the phase shift, *θ* = *π*, of the XKNL^56,57^. For analysis of the efficiency and performance of nonlinearly optical gates, based on the process model (Eqs 13 and 15) under the decoherence effect, we use commercial fibers^56,57^ with a signal loss of 0.364 dB/km (*χ*/*γ* = 0.0125) and pure silica core fibers^57^ with a signal loss of 0.15 dB/km (*χ*/*γ* = 0.0303), representing current technology.

### Path-parity gates (1 and 2)

When the path-parity gates (1 and 2) are implemented in an optical fiber^56,57^, we should consider how the decoherence effect (photon loss and dephasing) affects the efficiency and performance of the output states. Thus, the output states (${|{{\rm{\Phi }}}_{2}\rangle }_{{\rm{CAB}}\otimes {\rm{P}}}$ in Eq. 3 and ${|{{\rm{\Phi }}}_{4}\rangle }_{{\rm{CAB}}\otimes {\rm{P}}}$ in Eq. 7) of path-parity gates (1 and 2) will be modified into the form of a density matrix, as a result of the decoherence effect, as follows:

15 $${{\rho }}_{{\rm{CAB}}\otimes {\rm{P}}}^{2}={{\rho }}_{{\rm{CAB}}\otimes {\rm{P}}}^{4}=\frac{1}{4}(\begin{array}{llll}1 & {|{\rm{KC}}|}^{2} & {|{\rm{L}}|}^{2} & {|{\rm{OC}}|}^{2}\\ {|{\rm{KC}}|}^{2} & 1 & {|{\rm{OC}}|}^{2} & {|{\rm{L}}|}^{2}\\ {|{\rm{L}}|}^{2} & {|{\rm{OC}}|}^{2} & 1 & {|{\rm{MC}}|}^{2}\\ {|{\rm{OC}}|}^{2} & {|{\rm{L}}|}^{2} & {|{\rm{MC}}|}^{2} & 1\end{array}),$$

where we define the bases of ${{\rho }}_{{\rm{CAB}}\otimes {\rm{P}}}^{2}$ and ${{\rho }}_{{\rm{CAB}}\otimes {\rm{P}}}^{4}$ from top to bottom and left to right by the output state of Eqs 3 and 8, as follows:

16 $$\begin{array}{lll}{{\rho }}_{{\rm{CAB}}\otimes {\rm{P}}}^{2} & : & \{\frac{1}{\sqrt{2}}({|H\rangle }_{{\rm{C}}}^{1}+{|V\rangle }_{{\rm{C}}}^{2}){|\psi \rangle }_{{\rm{A}}}^{1}{|\phi \rangle }_{{\rm{B}}}^{1}\otimes {|{{\rm{\Lambda }}}_{t}^{2}\alpha \rangle }_{{\rm{P}}}^{{\rm{a}}}{|0\rangle }_{{\rm{P}}}^{{\rm{b}}},\\  &  & \frac{1}{\sqrt{2}}({|H\rangle }_{{\rm{C}}}^{1}+{|V\rangle }_{{\rm{C}}}^{2}){|\psi \rangle }_{{\rm{A}}}^{2}{|\phi \rangle }_{{\rm{B}}}^{2}\otimes {|{{\rm{\Lambda }}}_{t}^{2}\alpha \rangle }_{{\rm{P}}}^{{\rm{a}}}{|0\rangle }_{{\rm{P}}}^{{\rm{b}}},\\  &  & \frac{1}{\sqrt{2}}({|H\rangle }_{{\rm{C}}}^{1}+{|V\rangle }_{{\rm{C}}}^{2}){|\psi \rangle }_{{\rm{A}}}^{1}{|\phi \rangle }_{{\rm{B}}}^{2}\otimes {|{{\rm{\Lambda }}}_{t}^{2}\alpha \,\cos \,{\rm{\theta }}\rangle }_{{\rm{P}}}^{{\rm{a}}}{|i{{\rm{\Lambda }}}_{t}^{2}\alpha \,\sin \,{\rm{\theta }}\rangle }_{{\rm{P}}}^{{\rm{b}}},\\  &  & \frac{1}{\sqrt{2}}({|H\rangle }_{{\rm{C}}}^{1}+{|V\rangle }_{{\rm{C}}}^{2}){|\psi \rangle }_{{\rm{A}}}^{2}{|\phi \rangle }_{{\rm{B}}}^{1}\otimes {|{{\rm{\Lambda }}}_{t}^{2}\alpha \,\cos \,{\rm{\theta }}\rangle }_{{\rm{P}}}^{{\rm{a}}}{|-i{{\rm{\Lambda }}}_{t}^{2}\alpha \,\sin \,{\rm{\theta }}\rangle }_{{\rm{P}}}^{{\rm{b}}}\},\\ {{\rho }}_{{\rm{CAB}}\otimes {\rm{P}}}^{4} & : & \{{|H\rangle }_{{\rm{C}}}^{1}{|\psi \rangle }_{{\rm{A}}}^{1}{|\phi \rangle }_{{\rm{B}}}^{1}\otimes {|{{\rm{\Lambda }}}_{t}^{2}\alpha \rangle }_{{\rm{P}}}^{{\rm{a}}}{|0\rangle }_{{\rm{P}}}^{{\rm{b}}},\\  &  & {|V\rangle }_{{\rm{C}}}^{2}{|\phi \rangle }_{{\rm{A}}}^{2}{|\psi \rangle }_{{\rm{B}}}^{2}\otimes {|{{\rm{\Lambda }}}_{t}^{2}\alpha \rangle }_{{\rm{P}}}^{{\rm{a}}}{|0\rangle }_{{\rm{P}}}^{{\rm{b}}},\\  &  & {|H\rangle }_{{\rm{C}}}^{1}{|\phi \rangle }_{{\rm{A}}}^{2}{|\psi \rangle }_{{\rm{B}}}^{2}\otimes {|{{\rm{\Lambda }}}_{t}^{2}\alpha \,\cos \,{\rm{\theta }}\rangle }_{{\rm{P}}}^{{\rm{a}}}{|i{{\rm{\Lambda }}}_{t}^{2}\alpha \,\sin \,{\rm{\theta }}\rangle }_{{\rm{P}}}^{{\rm{b}}},\\  &  & {|V\rangle }_{{\rm{C}}}^{2}{|\psi \rangle }_{{\rm{A}}}^{1}{|\phi \rangle }_{{\rm{B}}}^{1}\otimes {|{{\rm{\Lambda }}}_{t}^{2}\alpha \,\cos \,{\rm{\theta }}\rangle }_{{\rm{P}}}^{{\rm{a}}}{|-i{{\rm{\Lambda }}}_{t}^{2}\alpha \,\sin \,{\rm{\theta }}\rangle }_{{\rm{P}}}^{{\rm{b}}}\},\end{array}$$

where Λ = *e*^−*γt*/2^ is the rate of remaining photons resulting from photon loss. Regarding the above equations (15 and 16), the forms of the two output states (${|{{\rm{\Phi }}}_{2}\rangle }_{{\rm{CAB}}\otimes {\rm{P}}}$ and ${|{{\rm{\Phi }}}_{4}\rangle }_{{\rm{CAB}}\otimes {\rm{P}}}$) are identical, Eq. 15, but have different basis sets, Eq. 16. Also, using the process model (Eq. 14), the coherent parameters (C, O, L, K, and M) in Eq. 15 are given by

17 $$\begin{array}{rcl}{\rm{C}} & = & \exp [-\frac{{\alpha }^{2}}{2}(1-{e}^{-\gamma {\rm{\Delta }}t})\sum _{n=1}^{N}{e}^{-\gamma \Delta t(n-1)}(1-{e}^{in{\rm{\Delta }}\theta })],\\ {\rm{O}} & = & \exp [-\frac{{\alpha }^{2}}{2}{e}^{-\gamma t}(1-{e}^{-\gamma {\rm{\Delta }}t})(1-{e}^{i\theta })\sum _{n=1}^{N}{e}^{-\gamma {\rm{\Delta }}t(n-1)}]\\ {\rm{L}} & = & \exp [-\frac{{\alpha }^{2}}{2}{e}^{-\gamma t}(1-{e}^{-\gamma {\rm{\Delta }}t})\sum _{n=1}^{N}{e}^{-\gamma {\rm{\Delta }}t(n-1)}(1-{e}^{in{\rm{\Delta }}\theta })],\\ {\rm{M}} & = & \exp [-\frac{{\alpha }^{2}}{2}{e}^{-\gamma t}(1-{e}^{-\gamma {\rm{\Delta }}t})\sum _{n=1}^{N}{e}^{-\gamma {\rm{\Delta }}t(n-1)}(1-{e}^{i(\theta +n{\rm{\Delta }}\theta )})],\\ {\rm{K}} & = & \exp [-\frac{{\alpha }^{2}}{2}{e}^{-\gamma t}(1-{e}^{-\gamma {\rm{\Delta }}t})\sum _{n=1}^{N}{e}^{-\gamma {\rm{\Delta }}t(n-1)}(1-{e}^{i(\theta -n{\rm{\Delta }}\theta )})],\end{array}$$

where ${\tilde{D}}_{t}{\tilde{X}}_{t}={({\tilde{D}}_{{\rm{\Delta }}t}{\tilde{X}}_{{\rm{\Delta }}t})}^{N}$, and *θ* = *χt* = *χN*Δ*t* = *N*Δ*θ* for small time, Δ*t* (=*t*/*N*), and *α* ∈ **R**. We can quantify the degree of dephasing to evolve a pure state into a mixed state using the coherent parameters in Eq. 15.

**Figure 5:**
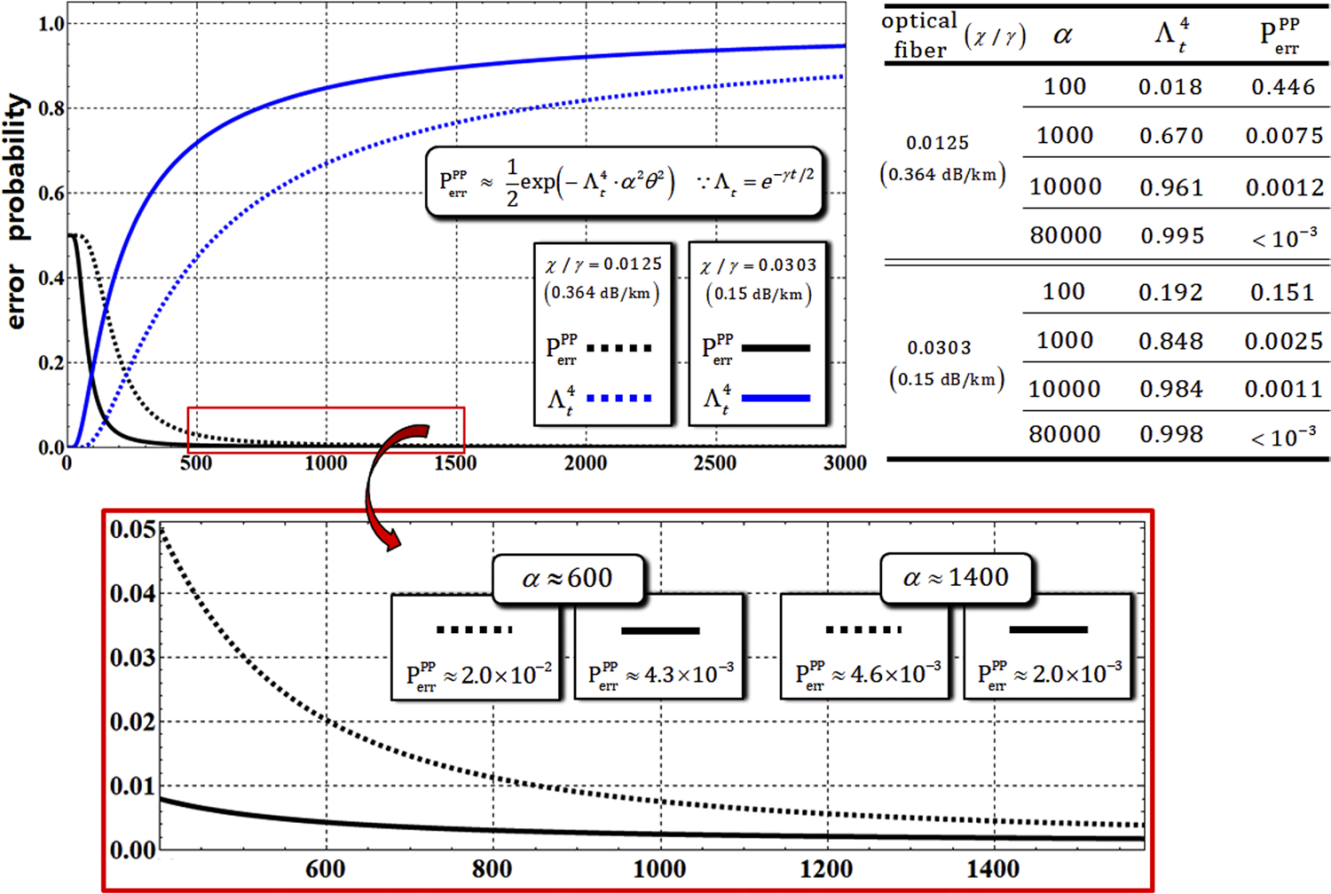
Graph represents the modified error probability, ${{\rm{P}}}_{{\rm{err}}}^{{\rm{PP}}}$, and the rate of remaining photons, ${{\rm{\Lambda }}}_{t}^{4}$ in path-parity gates (1 and 2) for *αθ* = 2.5, with optical fibers having signal losses of 0.364 dB/km (*χ*/*γ* = 0.0125) and 0.15 dB/km (*χ*/*γ* = 0.0303). In the other graph (red box), the values and plots of error probabilities depending on optical fibers are expressed for the range of the amplitude of the coherent state (500 < *α* < 1500). Also, the values of the error probabilities and the rates of remaining photons are provided in the Table for the difference in amplitude of coherent states with *αθ* = 2.5.

First, for the analysis of the efficiency of the path-parity gate, we fix the parameter value, *αθ* = *αχt* = 2.5, for ${{\rm{P}}}_{{\rm{err}}}^{{\rm{P}}} < {10}^{-3}$ (which is the error probability, Eq. 11, without the decoherence effect), and assume that the path-parity gate is operated in optical fibers^56,57^ having signal losses of 0.364 dB/km (*χ*/*γ* = 0.0125) and 0.15 dB/km (*χ*/*γ* = 0.0303). Figure 5 represents the modified error probability, ${{\rm{P}}}_{{\rm{err}}}^{{\rm{PP}}}$, of the output state, ${{\rho }}_{{\rm{CAB}}\otimes {\rm{P}}}^{2}$ or ${{\rho }}_{{\rm{CAB}}\otimes {\rm{P}}}^{{\rm{4}}}$, and the rate, ${{\rm{\Lambda }}}_{t}^{4}$, of the remaining photons in the probe beam against the decoherence effect caused by optical fibers having signal losses of 0.364 dB/km (*χ*/*γ* = 0.0125) and 0.15 dB/km (*χ*/*γ* = 0.0303). Because of the decoherence effect, the error probability, ${{\rm{P}}}_{{\rm{err}}}^{{\rm{PP}}}$, of the output state, ${{\rho }}_{{\rm{CAB}}\otimes {\rm{P}}}^{{\rm{2}}}$ or ${{\rho }}_{{\rm{CAB}}\otimes {\rm{P}}}^{4}$, is modified to

18 $$\begin{array}{l}{{\rm{P}}}_{{\rm{err}}}^{{\rm{PP}}}\approx \exp [-{{\rm{\Lambda }}}_{t}^{4}\cdot {\alpha }^{2}{\theta }^{2}]/2=\exp [-{e}^{-2\gamma t}\cdot {\alpha }^{2}{\theta }^{2}]/2,\\ \because \chi t=2.5/\alpha ,\chi /\gamma =0.0125({\rm{or}}\,0.0303)\end{array}$$

where Λ = *e*^−*γt*/2^ (the rate of remaining photons) with *αθ* = *αχt* = 2.5, and the signal loss of 0.364 dB/km (*χ*/*γ* = 0.0125) and 0.15 dB/km (*χ*/*γ* = 0.0303), depending on the optical fibers^56,57^. When increasing the amplitude of the coherent state (probe beam), the error probability, ${{\rm{P}}}_{{\rm{err}}}^{{\rm{PP}}}$, can be decreased, and also the rate, ${{\rm{\Lambda }}}_{t}^{4}$, of remaining photons can approach 1 with reliable PNR measurement, as described in Fig. 5. In addition, the values of the rate, ${{\rm{\Lambda }}}_{t}^{4}$, of remaining photons and the error probability, ${{\rm{P}}}_{{\rm{err}}}^{{\rm{PP}}}$, with respect to the signal loss rates of optical fibers and the amplitude of coherent states (100 ≤ *α* ≤ 80000), are listed in the Table of Fig. 5.

Consequently, by our analysis (using the process model, Eq. 14), the values in the Table clearly show that the path-parity gate can obtain high efficiency, ${{\rm{P}}}_{{\rm{err}}}^{{\rm{PP}}} < {10}^{-3}$ and a high rate of remaining photons, ${{\rm{\Lambda }}}_{t}^{4}\to 1$, with fixed *αθ* = *αχt* = 2.5 in optical fibers when we employ a coherent state with a strong amplitude, *α* > 80000(probe beam) under the decoherence effect.

**Figure 6:**
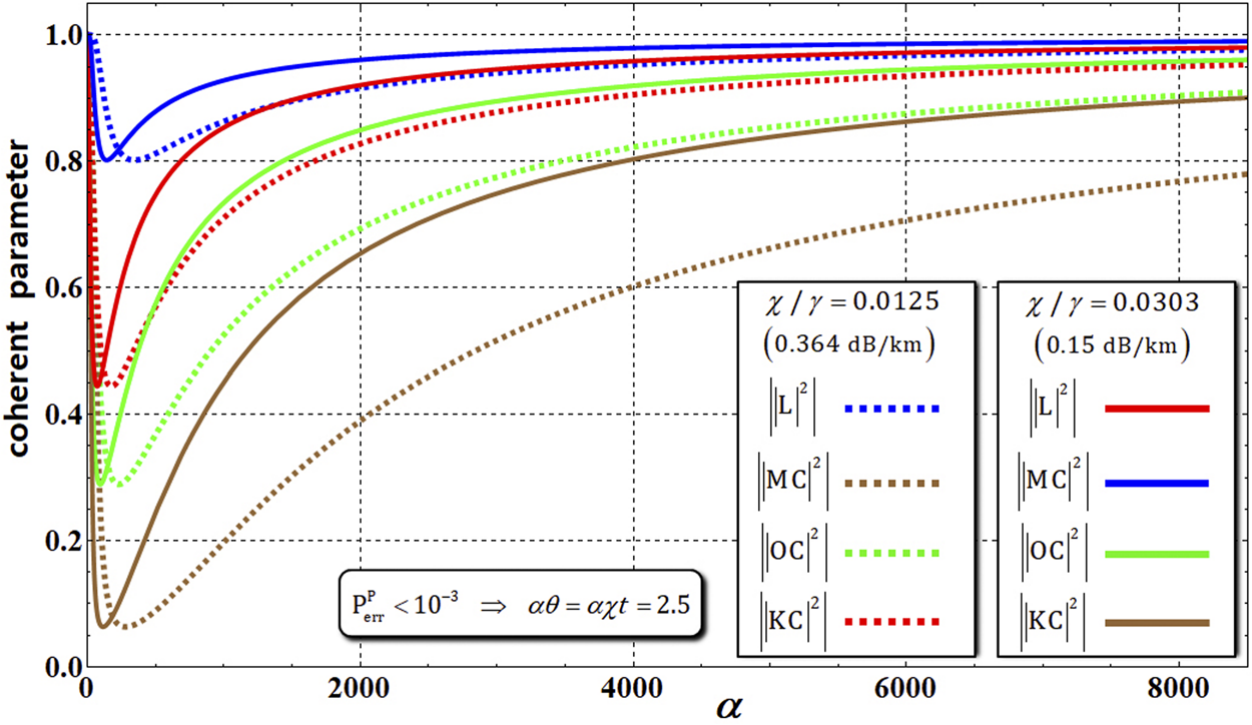
The graph represents the differences in the absolute values of coherent parameters (off-diagonal terms) in ${{\rho }}_{{\rm{CAB}}\otimes {\rm{P}}}^{2}$ and ${{\rho }}_{{\rm{CAB}}\otimes {\rm{P}}}^{4}$, according to the amplitude of the coherent state with *αθ* = *αχt* = 2.5 and *N* = 10^3^ in optical fibers^56,57^. Using our process model (Eq. 14), the absolute values of coherent parameters will approach 1 with increasing amplitude of the coherent state (*α* > 8000) in optical fibers with signal losses of 0.364 dB/km (*χ*/*γ* = 0.0125) and 0.15 dB/km (*χ*/*γ* = 0.0303).

Second, for analysis of the performance of the path-parity gate under the decoherence effect, we should consider the values of coherent parameters, which can quantify the amount of evolution of the pure state into the mixed state, in Eq. 15, and we also should calculate the fidelities between the density matrices (${{\rho }}_{{\rm{CAB}}\otimes {\rm{P}}}^{2}$ and ${{\rho }}_{{\rm{CAB}}\otimes {\rm{P}}}^{4}$ in Eq. 15) and output states (${|{{\rm{\Phi }}}_{2}\rangle }_{{\rm{CAB}}\otimes {\rm{P}}}$ in Eq. 3, and ${|{{\rm{\Phi }}}_{4}\rangle }_{{\rm{CAB}}\otimes {\rm{P}}}$ in Eq. 7) in optical fibers^56,57^ having signal losses of 0.364 dB/km (*χ*/*γ* = 0.0125) and 0.15 dB/km (*χ*/*γ* = 0.0303). If we consider the ideal case (without the decoherence effect: the output states in Sec. 2), all of the absolute values of the off-diagonal terms in the output states, ${|{{\rm{\Phi }}}_{2}\rangle }_{{\rm{CAB}}\otimes {\rm{P}}}$ and ${|{{\rm{\Phi }}}_{4}\rangle }_{{\rm{CAB}}\otimes {\rm{P}}}$ (i.e., $|{{\rm{\Phi }}}_{2}\rangle {\langle {{\rm{\Phi }}}_{2}|}_{{\rm{CAB}}\otimes {\rm{P}}}$ and $|{{\rm{\Phi }}}_{4}\rangle {\langle {{\rm{\Phi }}}_{4}|}_{{\rm{CAB}}\otimes {\rm{P}}}$: the form of the density matrix), of the path-parity gates are 1. This means that the output states are maintained in the pure states. However, the nonlinearly optical gates cannot avoid the decoherence effect when they are implemented in practice. This effect finally induces the pure state to evolve into the mixed state (classical state) by the dephasing of coherent parameters. To analyze this process, we apply the forms of density matrices (${{\rho }}_{{\rm{CAB}}\otimes {\rm{P}}}^{2}$ and ${{\rho }}_{{\rm{CAB}}\otimes {\rm{P}}}^{4}$ in Eq. 15), which consider the coherent parameters by dephasing via our process model, Eq. 14. As described in Fig. 6, the absolute values of coherent parameters (the off-diagonal terms, |KC|^2^, |OC|^2^, |MC|^2^, and |L|^2^ in ${{\rho }}_{{\rm{CAB}}\otimes {\rm{P}}}^{2}$ and ${{\rho }}_{{\rm{CAB}}\otimes {\rm{P}}}^{4}$) will approach 1 according to our the process model (Eq. 14) with increasing amplitude of the coherent state (probe beams) for *αθ* = *αχt* = 2.5 and *N* = 10^3^ (for a good approximation) in optical fibers^56,57^ having signal losses of 0.364 dB/km (*χ*/*γ* = 0.0125) and 0.15 dB/km (*χ*/*γ* = 0.0303). Finally, in Fig. 6, when the path-parity gates are experimentally implemented in optical fibers, we can retain the output states as pure states (the absolute values of coherent parameters are 1) by utilizing the strong amplitude of the coherent state with fixed *αθ* = *αχt* = 2.5 and *N* = 10^3^.

**Figure 7:**
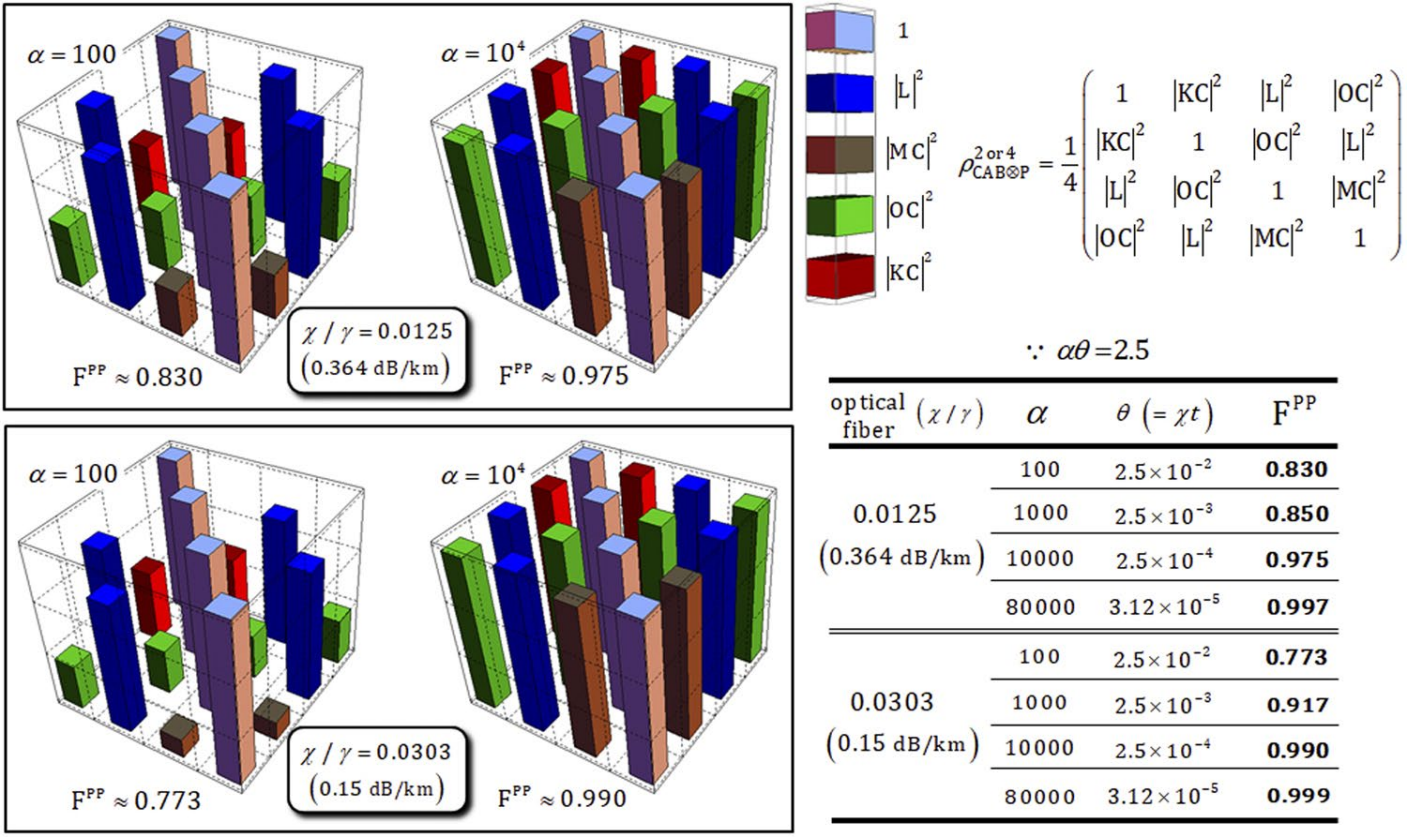
With the fixed parameters *αθ* = *αχt* = 2.5 and *N* = 10^3^ in optical fibers (with signal losses of 0.364 dB/km and 0.15 dB/km), the fidelities, F^PP^, of the output states in path-parity gates are represented in diagrams depending on the amplitudes of the coherent state (*α* = 100, 10^4^). The values of fidelities and magnitude of conditional phase shifts are shown in the Table as calculated using our process model (Eq. 14) with *αθ* = *αχt* = 2.5 and *N* = 10^3^ in optical fibers. If the amplitude of the coherent state increases, the fidelities increase (F^PP^ → 1) and the magnitude of conditional phase shifts decrease (*θ* = *χt* → *s*mall), indicating reliable performance of the path-parity gates.

Figure 7 shows the diagrams of the values of coherent parameters in ${{\rho }}_{{\rm{CAB}}\otimes {\rm{P}}}^{2}$ and ${{\rho }}_{{\rm{CAB}}\otimes {\rm{P}}}^{4}$ (shortly ${{\rho }}_{{\rm{CAB}}\otimes {\rm{P}}}^{2\,{\rm{or}}\,4}$) and fidelities (F^PP^), according to the amplitude (*α* = 100, 10^4^) of the coherent state with *αθ* = *αχt* = 2.5 and *N* = 10^3^ in optical fibers having signal losses of 0.364 dB/km (*χ*/*γ* = 0.0125) and 0.15 dB/km (*χ*/*γ* = 0.0303). The fidelity, F^PP^, between the output states (${|{{\rm{\Phi }}}_{2}\rangle }_{{\rm{CAB}}\otimes {\rm{P}}}$ and ${|{{\rm{\Phi }}}_{4}\rangle }_{{\rm{CAB}}\otimes {\rm{P}}}$ without the decoherence effect) and the density matrices (${{\rho }}_{{\rm{CAB}}\otimes {\rm{P}}}^{2\,{\rm{or}}\,4}$: under the decoherence effect) is given by

19 $${{\rm{F}}}^{{\rm{PP}}}\equiv |\sqrt{\langle {{\rm{\Phi }}}_{2}|{{\rho }}_{{\rm{CAB}}\otimes {\rm{P}}}^{2}|{{\rm{\Phi }}}_{2}\rangle }|=|\sqrt{\langle {{\rm{\Phi }}}_{4}|{{\rho }}_{{\rm{CAB}}\otimes {\rm{P}}}^{4}|{{\rm{\Phi }}}_{4}\rangle }|=\frac{1}{2}|\sqrt{1+{|{\rm{L}}|}^{2}+|{\rm{OC}}{|}^{2}+(|{\rm{KC}}{|}^{2}+|{\rm{MC}}{|}^{2})/2}|,$$

where C, O, L, K, and M are the coherent parameters in Eq. 17. As described in Fig. 7, we can confirm the high fidelities (F^PP^ > 0.9) of the output states when utilizing the strong amplitude of the coherent state (*α* > 10^4^). The various values of fidelities and the required magnitude of conditional phase shifts (*θ* = *χt*), according to the amplitudes of the coherent state with *αθ* = *αχt* = 2.5 and *N* = 10^3^, are summarized in the Table in Fig. 7. From this result (using the strong coherent state), we can obtain two advantages for reliable performance of path-parity gates: (1) high fidelity – According to our process model, the coherent parameters in output states, ${{\rho }}_{{\rm{CAB}}\otimes {\rm{P}}}^{2\,{\rm{or}}\,4}$, approach 1 to maintain pure states. Specifically, we can avoid the evolution into mixed states induced by dephasing of coherent parameters; (2) feasible implementation – The magnitude of the conditional phase shift in nature is tiny, *θ* ≈ 10^−18 ^^59^, although it can be increased by electromagnetically induced transparency, *θ* ≈ 10^−2 ^^43,60^. By our analysis, the magnitude of the conditional phase shift is required to be small with fixed *αθ* = *αχt* = 2.5 and *N* = 10^3^ when increasing the amplitude of the coherent state (i.e., if *α* = 80000 in the optical fiber with signal loss of 0.15 dB/km, then F^PP^~0.999 and *θ* ~ 3.12 × 10^−5^, as listed in the Table of Fig. 7). Thus, when we employ the strong coherent state (probe beam), path-parity gates are feasible to experimentally realize in practice because of the small conditional phase shift.

### Path-merging gates (1 and 2)

We should also consider the effect of decoherence in the path-merging gates (1 and 2) on the efficiency and performance of the output states. The output states (${|{{\rm{\Phi }}}_{6}\rangle }_{{\rm{ABC}}\otimes {\rm{P}}}$ in Eq. 7, and ${|{{\rm{\Phi }}}_{7}\rangle }_{{\rm{ABC}}\otimes {\rm{P}}}$ in Eq. 8) of the path-merging gates (1 and 2) should be modified by the decoherence effect as follows:

20 $${{\rho }}_{{\rm{ABC}}\otimes {\rm{P}}}^{6}={{\rho }}_{{\rm{ABC}}\otimes {\rm{P}}}^{7}=\frac{1}{2}(\begin{array}{cc}1 & |{\rm{C}}{|}^{2}\\ |{\rm{C}}{|}^{2} & 1\end{array}),$$

where we define the bases of ${{\rho }}_{{\rm{ABC}}\otimes {\rm{P}}}^{6}$ and ${{\rho }}_{{\rm{ABC}}\otimes {\rm{P}}}^{7}$ from top to bottom and left to right by the output state of Eqs 8 and 9, as follows:

21 $$\begin{array}{ccc}{\rho }_{{\rm{A}}{\rm{B}}{\rm{C}}\otimes {\rm{P}}}^{6} & : & \{\frac{1}{\sqrt{2}}({|\psi \rangle }_{{\rm{A}}}^{1}{|\phi \rangle }_{{\rm{B}}}^{1}{|H\rangle }_{{\rm{C}}}^{1}+{|\phi \rangle }_{{\rm{A}}}^{1}{|\psi \rangle }_{{\rm{B}}}^{2}{|V\rangle }_{{\rm{C}}}^{1})\otimes {|\alpha \rangle }_{{\rm{P}}}^{{\rm{a}}}{|0\rangle }_{{\rm{P}}}^{{\rm{b}}},\\  &  & \frac{1}{\sqrt{2}}({|\psi \rangle }_{{\rm{A}}}^{2}{|\phi \rangle }_{{\rm{B}}}^{1}{|H\rangle }_{{\rm{C}}}^{1}-{|\phi \rangle }_{{\rm{A}}}^{2}{|\psi \rangle }_{{\rm{B}}}^{2}{|V\rangle }_{{\rm{C}}}^{1})\otimes {|{{\rm{\Lambda }}}_{t}\alpha \,\cos \,\theta \rangle }_{{\rm{P}}}^{{\rm{a}}}{|i\,{{\rm{\Lambda }}}_{t}\alpha \,\sin \,\theta \rangle }_{{\rm{P}}}^{{\rm{b}}}\},\\ {\rho }_{{\rm{A}}{\rm{B}}{\rm{C}}\otimes {\rm{P}}}^{7} & : & \{\frac{1}{\sqrt{2}}({|\psi \rangle }_{{\rm{A}}}^{1}{|\phi \rangle }_{{\rm{B}}}^{1}{|H\rangle }_{{\rm{C}}}^{1}+{|\phi \rangle }_{{\rm{A}}}^{1}{|\psi \rangle }_{{\rm{B}}}^{1}{|V\rangle }_{{\rm{C}}}^{1})\otimes {|\alpha \rangle }_{{\rm{P}}}^{{\rm{a}}}{|0\rangle }_{{\rm{P}}}^{{\rm{b}}},\\  &  & \frac{1}{\sqrt{2}}({|\psi \rangle }_{{\rm{A}}}^{1}{|\phi \rangle }_{{\rm{B}}}^{2}{|H\rangle }_{{\rm{C}}}^{1}-{|\phi \rangle }_{{\rm{A}}}^{1}{|\psi \rangle }_{{\rm{B}}}^{2}{|V\rangle }_{{\rm{C}}}^{1})\otimes {|{{\rm{\Lambda }}}_{t}\alpha \,\cos \,\theta \rangle }_{{\rm{P}}}^{{\rm{a}}}{|i{{\rm{\Lambda }}}_{t}\alpha \,\sin \,\theta \rangle }_{{\rm{P}}}^{{\rm{b}}}\}.\end{array}$$

Regarding these equations (20 and 21), the two output states (${|{{\rm{\Phi }}}_{2}\rangle }_{{\rm{CAB}}\otimes {\rm{P}}}$ and ${|{{\rm{\Phi }}}_{4}\rangle }_{{\rm{CAB}}\otimes {\rm{P}}}$) have the same form (density matrix, Eq. 20) while having different basis sets, Eq. 21. In density matrices, ${{\rho }}_{{\rm{A}}{\rm{B}}{\rm{C}}\otimes {\rm{P}}}^{6}$ and ${{\rho }}_{{\rm{ABC}}\otimes {\rm{P}}}^{7}$, the coherent parameter, C, which can quantify the dephasing, is given in Eq. 17, where *θ* = *χt* = *χN*Δ*t* = *N*Δ*θ* for small time, Δ*t* (=*t*/*N*), and *α* ∈ **R**.

**Figure 8:**
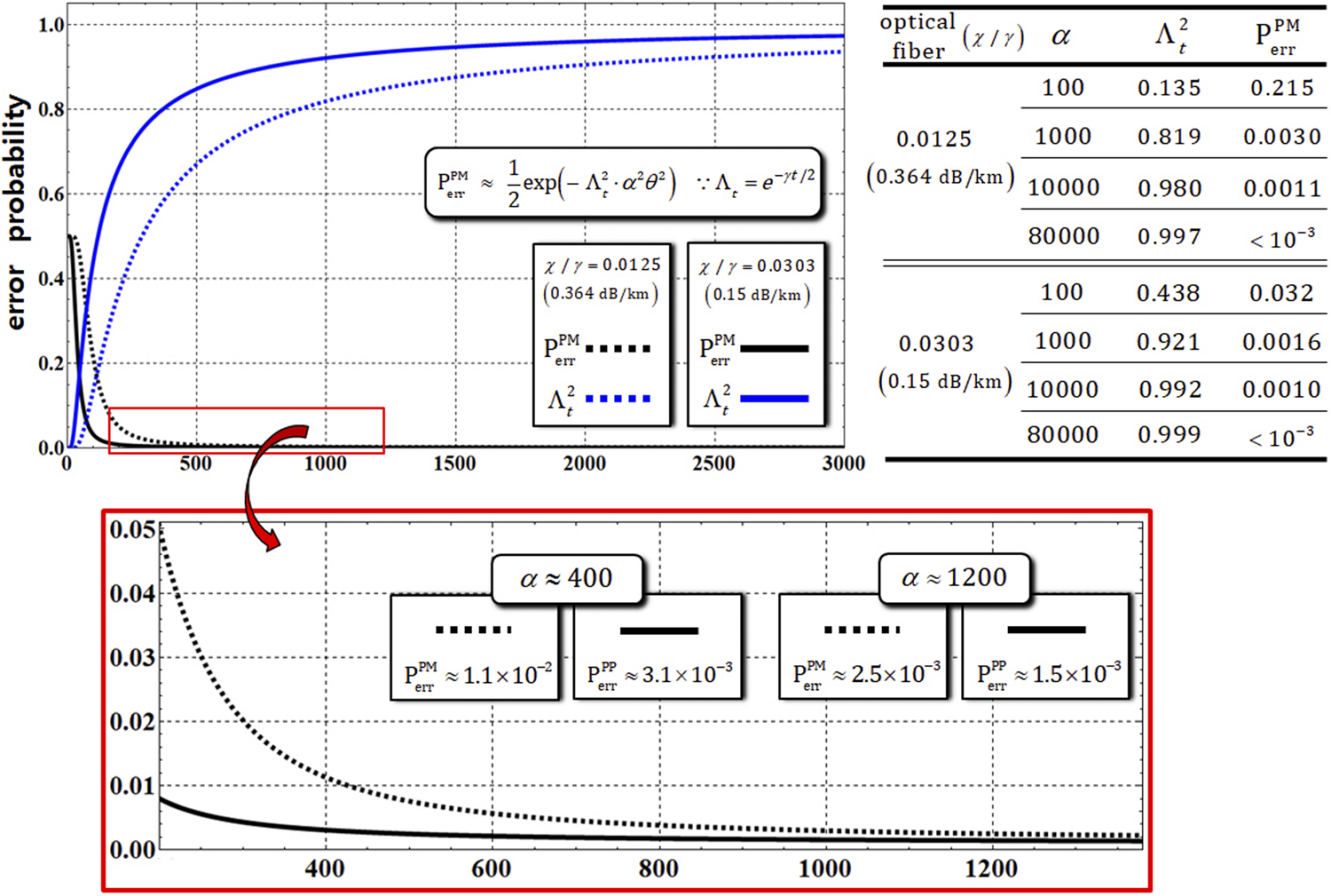
Graph represents the modified error probability, ${{\rm{P}}}_{{\rm{err}}}^{{\rm{PM}}}$, and the rate of remaining photons, ${{\rm{\Lambda }}}_{t}^{2}$ in path-merging gates (1 and 2) for *αθ* = 2.5 with optical fibers having signal losses of 0.364 dB/km (*χ*/*γ* = 0.0125) and 0.15 dB/km (*χ*/*γ* = 0.0303). In the other graph (red box), the values and plots of error probabilities depending on optical fibers are expressed in the range of amplitude of the coherent state (300 < *α* < 1300). Also, the values of the error probabilities and the rates of remaining photons are provided in the Table for the difference in amplitude of the coherent states with *αθ* = 2.5.

First, for the analysis of the efficiency of the path-merging gate, comparing the error probability, ${{\rm{P}}}_{{\rm{err}}}^{{\rm{PM}}}$ in Eq. 11, without the decoherence effect, we should recalculate the error probability, ${{\rm{P}}}_{{\rm{err}}}^{{\rm{PM}}}$, of the output state, ${{\rho }}_{{\rm{ABC}}\otimes {\rm{P}}}^{6}$ and ${{\rho }}_{{\rm{ABC}}\otimes {\rm{P}}}^{7}$ including photon loss, as follows:

22 $$\begin{array}{c}{{\rm{P}}}_{{\rm{e}}{\rm{r}}{\rm{r}}}^{{\rm{P}}{\rm{M}}}\approx \exp [\,-{{\rm{\Lambda }}}_{t}^{2}\cdot {\alpha }^{2}{\theta }^{2}]/2=\exp [\,-{e}^{-\gamma t}\cdot {\alpha }^{2}{\theta }^{2}]/2,\\ \because \chi t=2.5/\alpha ,\,\chi /\gamma =0.0125\,({\rm{o}}{\rm{r}}\,0.0303)\end{array}$$

where ${{\rm{\Lambda }}}_{t}={e}^{-\gamma t/2}$ (the rate of remaining photons) with *αθ* = *αχt* = 2.5, and signal losses of 0.364 dB/km (*χ*/*γ* = 0.0125) and 0.15 dB/km (*χ*/*γ* = 0.0303), depending on the optical fibers^56,57^. In Fig. 8 and the Table therein, as the amplitude of the coherent state in path-merging gates increases, we can confirm the decreasing error probability, ${{\rm{P}}}_{{\rm{err}}}^{{\rm{PM}}}\to 0$, and the increasing rate of remaining photons, ${{\rm{\Lambda }}}_{t}^{2}\to 1$. Consequently, as with the path-parity gates (1 and 2), the values in the Table in Fig. 8 show that high efficiency, ${{\rm{P}}}_{{\rm{err}}}^{{\rm{PM}}} < {10}^{-3}$ and a high rate of remaining photons ${{\rm{\Lambda }}}_{t}^{2}\to 1$, with fixed *αθ* = *αχt* = 2.5 in optical fibers can be acquired, through our analysis (Eq. 14), using a coherent state with strong amplitude, *α* > 80000 (probe beam), under the decoherence effect.

**Figure 9:**
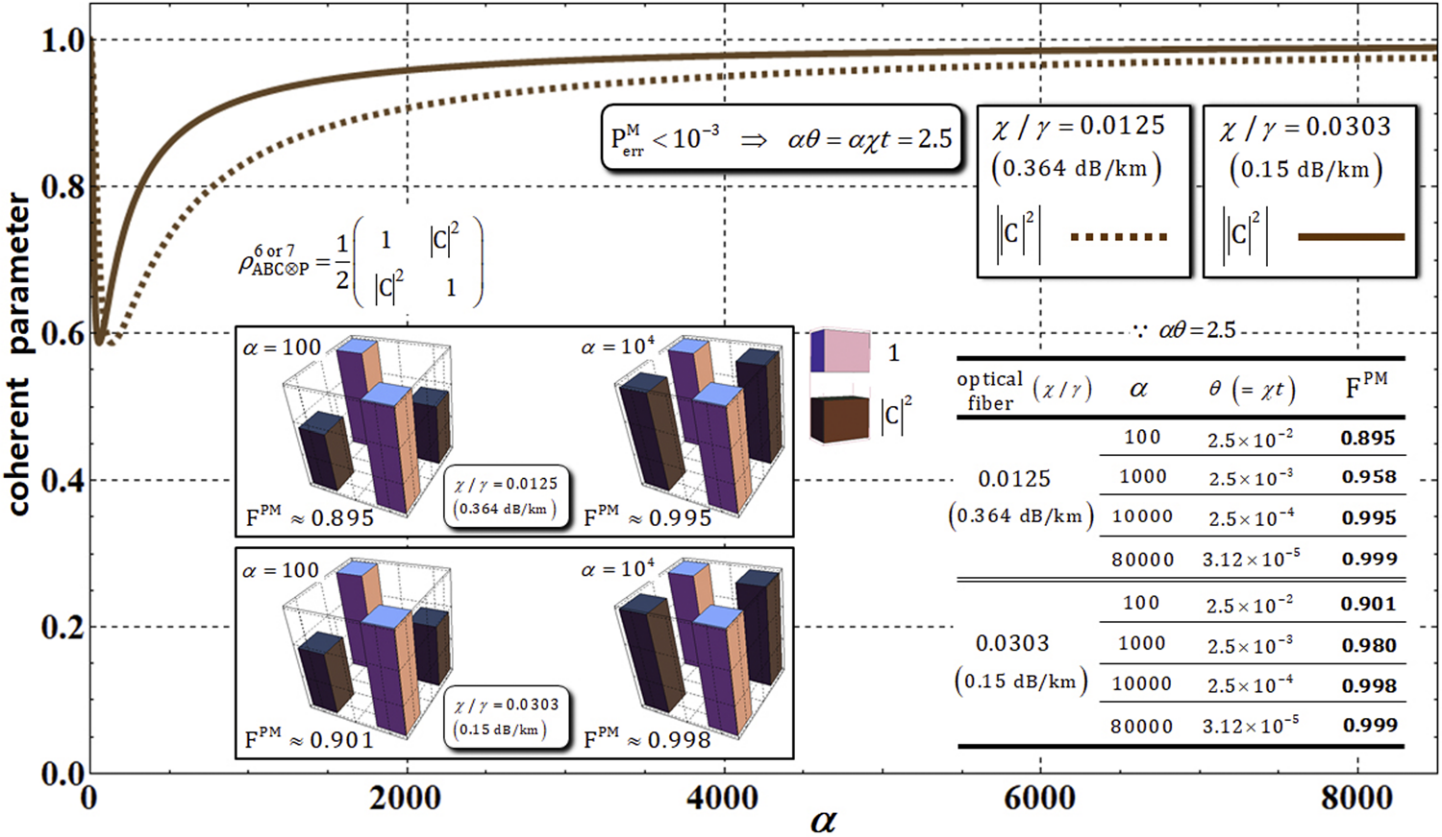
The graph represents the absolute value of the coherent parameter (off-diagonal terms) in ${{\rho }}_{{\rm{ABC}}\otimes {\rm{P}}}^{6\,{\rm{or}}\,7}$ for the amplitude of the coherent state with *αθ* = *αχt* = 2.5 and *N* = 10^3^, with optical fibers^56,57^. The absolute value of the coherent parameter, |C|^2^, approaches 1 if the amplitude of the coherent state increases (*α* > 8000) according to the process model, Eq. 14. In diagrams and the Table, according to the amplitudes (100 ≤ *α* ≤ 80000) of the coherent state with fixed parameters, *αθ* = *αχt* = 2.5 and *N* = 10^3^, the differences in the absolute values of the coherent parameter and fidelities, F^PM^, are expressed in optical fibers. When increasing the amplitude of the coherent state, the fidelities increase (F^PM^ → 1) and the magnitude of conditional phase shifts decreases (*θ* = *χt* → *s*mall), indicating the reliable performance of the path-merging gates.

Second, for the analysis of the performance of the path-merging gate under the decoherence effect, we should analyze the absolute value of the coherent parameter, |C|^2^, in ${{\rho }}_{{\rm{ABC}}\otimes {\rm{P}}}^{6}$ and ${{\rho }}_{{\rm{ABC}}\otimes {\rm{P}}}^{7}$ (shortly ${{\rho }}_{{\rm{ABC}}\otimes {\rm{P}}}^{6\,{\rm{or}}\,7}$), and the fidelities, F^PM^, in optical fibers^56,57^ having signal losses of 0.364 dB/km (*χ*/*γ* = 0.0125) and 0.15 dB/km (*χ*/*γ* = 0.0303). As described in Fig. 9, the absolute values of the coherent parameter, |C|^2^, increase to maintain the output states (${{\rho }}_{{\rm{ABC}}\otimes {\rm{P}}}^{6\,{\rm{or}}\,7}$) in pure states (elimination of dephasing) by the strong coherent state under the decoherence effect, in practice (optical fibers). This result, suggesting that a strong coherent state should be utilized for the reduction of dephasing, is the same as the result of path-parity gates by our analysis. Also, in the diagrams and Table of Fig. 9, the fidelity, F^PM^, of the density matrices (${{\rho }}_{{\rm{ABC}}\otimes {\rm{P}}}^{6\,{\rm{or}}\,7}$: under the decoherence effect) is calculated as

23 $${{\bf{F}}}^{{\rm{PM}}}\equiv |\sqrt{\langle {{\rm{\Phi }}}_{6}|{{\rho }}_{{\rm{ABC}}\otimes P}^{6}|{{\rm{\Phi }}}_{6}\rangle }|=|\sqrt{\langle {{\rm{\Phi }}}_{7}|{{\rho }}_{{\rm{ABC}}\otimes {\rm{P}}}^{7}|{{\rm{\Phi }}}_{7}\rangle }|=\frac{1}{\sqrt{2}}|\sqrt{1+{|C|}^{2}}|.$$

Finally, for the reliable performance (high fidelity, and weak XKNL: small magnitude of conditional phase shift) of path-merging gates, we should increase the amplitude of the coherent state for *αθ* = *αχt* = 2.5 and *N* = 10^3^ when experimentally implemented path-merging gates under the decoherence effect, as described in Fig. 9.

Consequently, according to our analysis (the process model based on the master equation), we demonstrate that the utilization of the strong (increasing amNplitude) coherent state in nonlinearly optical gates (path-parity and path-merging gates in our SWAP test) will bring about high efficiency (small error probabilities) and reliable performance (robustness: high fidelities, and feasibility: weak XKNLs) with respect to the decoherence effect.

## Conclusions

We presented an optical scheme for the SWAP test (controlled swap operation), via nonlinearly optical (path-parity and path-merging) gates and a linearly optical (HOM) gate, to definitely determine whether the difference between two unknown states in Sec. 2. We also demonstrated a method, which should utilize a strong coherent state according to our analysis, to obtain high efficiency (low error probability) and reliable performance (high fidelity) in nonlinearly optical gates under the decoherence effect, in Sec. 3. Therefore, the proposed scheme (SWAP test via weak XKNLs, qubus beams, and PNR measurements) has the following advantages:

1. When presented with the question of whether two unknown states are equal or not, the SWAP test can determine with certainty whether two unknown states are different in various QIP schemes (quantum communications: quantum authentication, quantum signature, and quantum computation: quantum machine learning, and Fredkin gate). Thus, we proposed a deterministic (determination of difference between two unknown states) and feasible (experimental implementation) scheme for the SWAP test using weak XKNLs, qubus beams, and PNR measurements.
2. In this paper, we demonstrated that nonlinearly optical (path-parity and path-merging) gates, which are designed using XKNLs, qubus beams, and PNR measurement, should employ a coherent state with a strong amplitude to obtain high efficiency (low error probability) and reliable performance (high fidelity) according to our analysis using the process model in Sec. 3. In the previous works^23,24,28–30^, which have proposed the various nonlinearly optical gates (including to path-parity and path-merging gates), for quantum information processing schemes, the affection of the decoherence effect, in practice, have been overlooked. Compared with these works^28–30^, we analyzed the decoherence effect by master equation, and derived the method, using strong coherent state, to reduce photon loss and dephasing (decoherence). Thus, when our scheme for the SWAP test is experimentally realized, it will be robust against the decoherence effect (photon loss and dephasing).
3. Through the analysis in Sec. 3, we showed that our scheme (nonlinearly optical gates) require the small magnitude of the conditional phase shift (*θ*), as described in Figs 7 and 9, because the conditional phase shift from Kerr media is extreme small^59^, and difficult to increase by electromagnetically induced transparency^43,60^. But our gates, compared with the former works^23,24,28–30^, can obtain the high efficiency and reliable performance with tiny magnitude of conditional phase shift by utilizing the strong coherent state (for the reduction of decoherence effect), according to our analysis in Sec. 3. Therefore, when we employ the strong coherent state (probe beam), path-parity and path-merging gates are feasible to experimentally realize in practice because of the small conditional phase shift.
4. In our scheme, the designed nonlinearly optical gates employ qubus beams and the strategy of PNR measurement. Therefore, we employed only positive conditional phase shifts (*θ*) by XKNL in path-parity and path-merging gates. Kok in ref.^61^. showed that it is generally not possible to change the sign of the conditional phase shift (−*θ*). Thus, our nonlinearly optical gates using only positive conditional phase shifts (*θ*) with qubus beams and PNR measurement are more feasible than other nonlinearly optical gates^26,27,32,39,40^ that use the negative conditional phase shift (−*θ*).
5. As for a minor issue, because PNR measurements are applied on the probe beam of path b in all nonlinearly optical gates, the probe beam of path a can be recycled for other nonlinearly optical gates (if desired) for a more efficient implementation.

Consequently, we demonstrate that our scheme for the SWAP test to determine whether the difference between unknown states, using weak XKNLs, qubus beams, and PNR measurements, can be experimentally realized and is immune to the decoherence effect in optical fibers.

## Supplementary information

APPENDICES
