# Supplementary material for: Implementation of SWAP test for two unknown states in photons via cross-Kerr nonlinearities under decoherence effect
Source: Sci Rep. 2019 Apr 16;9:6167. doi: 10.1038/s41598-019-42662-4 (PMC6468003; doi:10.1038/s41598-019-42662-4)
Supplement: Supplementary file 1 — APPENDICES [file 41598_2019_42662_MOESM1_ESM.docx]

**Title: Implementation of SWAP test for two unknown states in photons via cross-Kerr nonlinearities under decoherence effect**

Authors: Min-Sung Kang, Jino Heo, Seong-Gon Choi, Sung Moon, Sang-Wook Han

**APPENDIX (A)**

In practice, for high photon number resolution, we can apply the quantum non-demolition detection (QND) [28, 29, 32, 41] in nonlinearly optical (path-parity and path-merging) gates, as shown in Fig. A1,


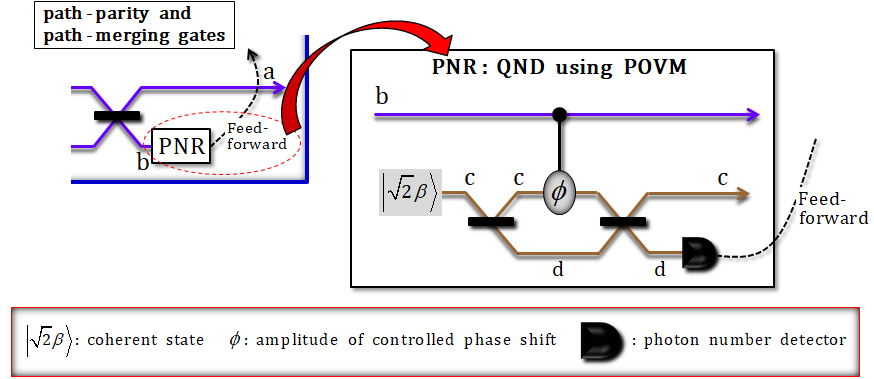


**Fig. A1.** For reliable PNR measurement of path b, the QND module [28, 29, 32, 41], which is consisted of the second probe beam, $|\sqrt{2}\beta\rangle$, two BSs, a controlled phase shift, , and a photon number detector of path d, can precisely distinguish the number of photons $|n\rangle_{\text{P}}^{\text{b}}$ in the probe beam of path b by positive-operator-value measurement (POVM) elements. And then, we can make a decision to operate the feed-forwards (PSs and switches), according to the result of the QND.

Figure A1 shows the operation of QND module to measure the photon number, $|n\rangle_{\text{P}}^{\text{b}}$, in the qubus (probe) beams ($|n\rangle_{\text{P}}^{\text{b}}$ or $|\pm i\alpha\sin\theta\rangle_{\text{P}}^{\text{b}}$ $∵n\neq0$) of path b. After operated the QND module (second probe beam, $|\sqrt{2}\beta\rangle_{\text{P}}^{\text{c}}$, and controlled phase shift, ) on path b of probe beam, the possible states (pre-measurement) on paths b, c, and d are transformed as

(A1)

where $n$ is photon number of probe beam on path b. This result means that probe beam of nonlinearly optical (path-parity and path-merging) gates on path b is coupled with the second probe beams on paths c and d by the QND module. For example, after the operation of QND, the output state, $|\Phi_{2}\rangle_{\text{CAB}\otimes\text{P}}$ in Eq. 3, of path-parity gate (1) will be coupled with the second probe beam on paths c and d, as follows:

(A2)

where for $\beta\in\mathbf{R}$. Due to the coupling between the qubus beam, $|0\rangle_{\text{P}}^{\text{b}}$ or $|n\rangle_{\text{P}}^{\text{b}}$, on path b and second probe beam, $|0\rangle_{\text{P}}^{\text{d}}$ or , on path d, we can distinguish the difference, small, in photon numbers on path b by utilizing the POVM elements [28, 29, 32, 41] to detect photon number of the state $|m\rangle_{\text{P}}^{\text{d}}$ on path d.


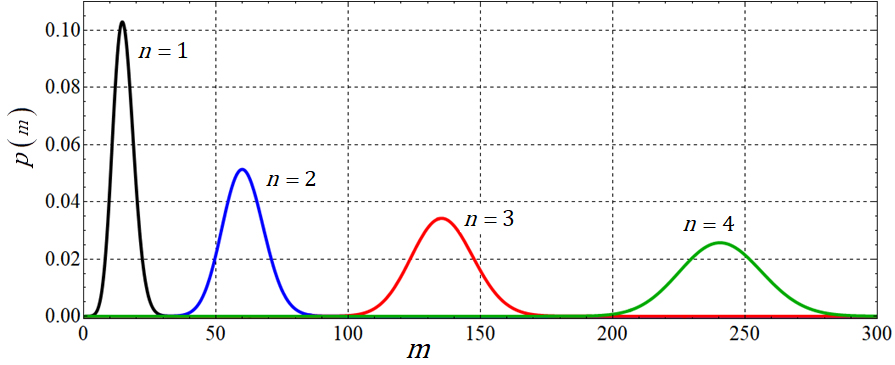


**Fig. A2.** This graph shows the Poisson distributions of the second probe beam, (coupled with probe beam, $|n\rangle_{\text{P}}^{\text{b}}$, on path b), on path d. And $m$ and are the photon number and the probability to detect the photon number,$m$, in the Poisson distribution of by POVM elements. When parameters (amplitude of second coherent state and magnitude of conditional phase shift) are $\beta=100$ and , the overlaps between photon numbers, $n$, on path b are negligible, according to the photon numbers, $m$, on path d in this QND module using POVM elements.

As described in Fig. A2, when the amplitude of second probe beam and magnitude of conditional phase shift in QND module are $\beta=100$ and , the small differences in photon numbers $|n\rangle_{\text{P}}^{\text{b}}\text{ }\left(n=1\text{ },\text{ }2\text{ },....\right)$ of qubus beam on path b can be discriminated from the photon number, $|m\rangle_{\text{P}}^{\text{d}}$, of by POVM elements [28, 29, 32, 41]. Consequently, the usage of QND module via POVM elements can be reliable implemented PNR measurement for $|n\rangle_{\text{P}}^{\text{b}}$ in qubus beam on path b.

**REFERENCES**

[28] Q. Lin and B. He, “Single-photon logic gates using minimal resources” Phys. Rev. A 80, 042310 (2009)

[29] Q. Lin, B. He, J. A. Bergou, and Y. Ren, “Processing multiphoton states through operation on a single photon: Methods and applications” Phys. Rev. A 80, 042311 (2009)

[32] J. Heo, C. H. Hong, H. J. Yang, J. P. Hong, and S. G. Choi, “Analysis of optical parity gates of generating Bell state for quantum information and secure quantum communication via weak cross-Kerr nonlinearity under decoherence effect” Quantum Inf. Process. 16, 10 (2017)

[41] J. Heo, C. H. Hong, D. H. Lee, and H. J. Yang, “Bidirectional transfer of quantum information for unknown photons via cross-Kerr nonlinearity and photon-number-resolving measurement” Chin. Phys. B 25, 020306 (2016)

**APPENDIX (B)**

Figure B1 schematically shows the operations of path switches $\text{S}$ and $\text{S}'$ in Figs. 3 and 4. The structure of path switches [28-31, 34, 42] is experimentally simple. According to outcomes of PNR measurement on path b of probe beam, the operation of feed-forward can be decided.


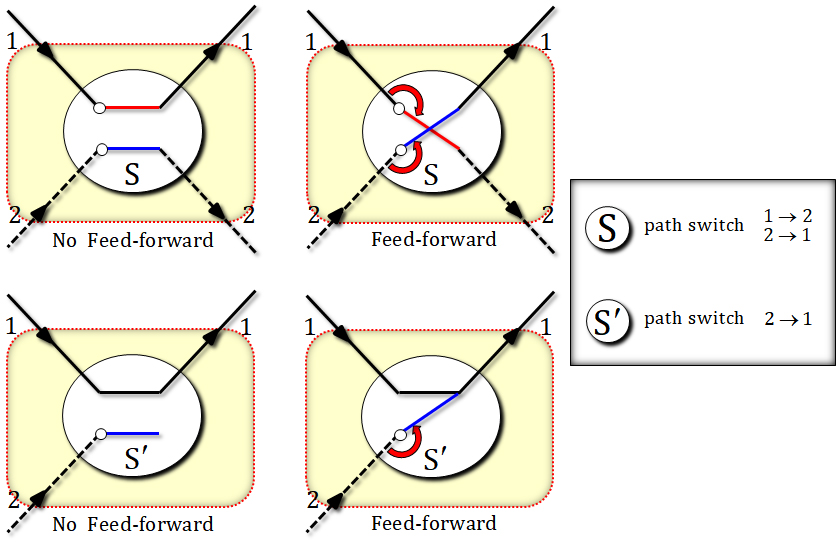


**Fig. B1.** Plot schematically represents the operations of path switches [28-31, 34, 42] in Figs. 3 and 4 in feed-forward or not.

If the outcome is in $n$ ($n\neq0$) of photon number in probe beam, feed-forward, including to path switch, is performed into output state to correct a phase (phase shifter), polarization (spin flipper), and path. Thus, in feed-forward, path switches, which have been employed in the former works [28-31, 34, 42], play the roles of changing path of output state, as described in Fig. B1.

**REFERENCES**

[28] Q. Lin and B. He, “Single-photon logic gates using minimal resources” Phys. Rev. A 80, 042310 (2009)

[29] Q. Lin, B. He, J. A. Bergou, and Y. Ren, “Processing multiphoton states through operation on a single photon: Methods and applications” Phys. Rev. A 80, 042311 (2009)

[30] Q. Lin and B. He, “Highly efficient processing of multi-photon states” Sci. Rep. 5, 12792 (2015)

[31] J. Heo, M. S. Kang, C. H. Hong, H. Yang, and S. G. Choi, “Discrete quantum Fourier transform using weak cross-Kerr nonlinearity and displacement operator and photon-number-resolving measurement under the decoherence effect” Quantum Inf. Process. 15, 4955 (2016)

[34] J. Heo, M. S. Kang, C. H. Hong, J. P. Hong, and S. G. Choi, “Preparation of quantum information encoded on three-photon decoherence-free states via cross-Kerr nonlinearities” Sci. Rep. 8, 13843 (2018)

[42] F. Wang, M. X. Luo, G. Xu, X. B. Chen, and Y. X. Yang, “Photonic quantum network transmission assisted by the weak cross-Kerr nonlinearity” Sci. Chin. Phys. Mech. Astro. 61, 060312 (2018)
